# Aging of Skeletal Muscle: From Molecular Mechanisms to Therapeutic Interventions

**DOI:** 10.1002/mco2.70995

**Published:** 2026-09-21

**Authors:** Ting Liu, Yaomin Hu

**Affiliations:** ^1^ Department of Geriatrics, Renji Hospital, School of Medicine Shanghai Jiaotong University Shanghai China

**Keywords:** molecular mechanisms, muscle health, sarcopenia, skeletal muscle aging, therapeutic intervention

## Abstract

Skeletal muscle aging is a major cause of frailty, metabolic dysfunction, and loss of independence in later life, yet it cannot be explained by muscle mass loss alone. Recent single‐cell, multi‐omics, and translational studies show that aged muscle is shaped by coordinated changes in myofibers, stem and stromal cells, immune and vascular compartments, neuromuscular control, and systemic metabolism. This review describes skeletal muscle aging as a gradual loss of tissue resilience rather than a simple decline in muscle mass. Impaired proteostasis, mitochondrial dysfunction, chronic inflammation, and cellular senescence are discussed as major, closely linked processes that contribute to defective regeneration, matrix remodeling, fibro‐adipogenic conversion and denervation, and are influenced by endocrine, metabolic, liver‐, adipose‐, gut‐, and brain‐derived signals. Current therapeutic evidence is reviewed with an emphasis on exercise and nutritional optimization as the clinical foundation, while mitochondrial, anabolic, senescence‐directed, and regenerative strategies are considered as emerging or investigational approaches. By bringing these findings together, this review highlights how studies of muscle aging can move beyond descriptive changes in mass and strength toward a clearer understanding of the biological processes that limit muscle function in later life.

## Graphical abstract

Skeletal muscle aging is driven by coordinated local breakdown across myofibers, stem and stromal cells, immune and vascular compartments, and neuromuscular control. These mechanisms promote mitochondrial dysfunction, inflammation, senescence, impaired regeneration, fibrosis, myosteatosis, denervation, and functional decline. Systemic endocrine‐metabolic stress and interorgan crosstalk further modify muscle aging and therapeutic responsiveness. Foundation care with exercise and nutrition, combined with mechanism‐matched interventions, may restore muscle resilience and preserve mobility during aging.

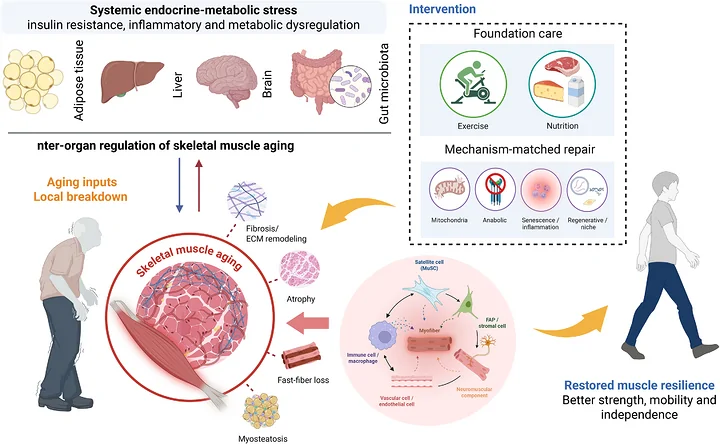

## 1 Introduction

Skeletal muscle aging is one of the most clinically visible manifestations of organismal aging because it directly affects mobility, metabolic health, independence, and resilience to disease [1, 2]. Unlike many molecular aging processes that remain clinically silent for long periods, deterioration of skeletal muscle is expressed through measurable declines in strength, gait speed, balance, endurance, and recovery from injury or illness [1, 2]. These functional consequences make muscle aging a major determinant of frailty, falls, disability, hospitalization, and loss of quality of life in older adults [1]. Skeletal muscle is also the largest metabolic organ in the body, and its deterioration influences glucose disposal, lipid metabolism, inflammatory tone, and whole‐body energy homeostasis [3]. Muscle aging therefore sits at the intersection of physical function, metabolic disease, and systemic aging [3, 4].

The clinical relevance of skeletal muscle aging is further highlighted by the frequent dissociation between muscle mass and muscle function [4, 5]. Strength, power, and mobility may decline more rapidly than muscle quantity, indicating that age‐related muscle impairment cannot be understood solely by measuring tissue mass [4]. Changes in muscle quality‐including fat infiltration, fibrosis, mitochondrial dysfunction, neuromuscular instability, vascular decline, and impaired regenerative capacity‐help explain why individuals with similar muscle mass may differ substantially in functional performance [4, 5]. Recent human cell atlases and multimodal studies reinforce this view by showing that aged muscle contains altered myofiber states, dysfunctional stem and stromal populations, immune and vascular remodeling, and disrupted intercellular communication rather than a uniform loss of contractile tissue [6, 7]. Together, these findings support a broader concept of skeletal muscle aging as a progressive decline in tissue resilience, here referring to the ability of muscle to preserve homeostasis, repair damage, and recover function after physiological or pathological stress [4, 6, 7].

Sarcopenia has provided an essential clinical framework for recognizing age‐related loss of muscle mass and function. However, the field is moving beyond a narrow disease definition toward the more integrative concept of muscle health [4, 8]. This shift is necessary because sarcopenia, as currently operationalized, often relies on thresholds for appendicular lean mass, grip strength, or physical performance, whereas the biology underlying functional decline is far more heterogeneous [4, 9]. Some older adults primarily exhibit inactivity‐related atrophy and anabolic resistance; others show prominent inflammatory remodeling, mitochondrial impairment, fibrosis, denervation, metabolic disease, or systemic endocrine disturbance. A single diagnostic label may therefore group together biologically distinct trajectories [10, 11, 12].

This changing view has been driven by advances in single‐cell, single‐nucleus, spatially informed, proteomic, metabolomic, and epigenomic approaches. These technologies show that skeletal muscle aging is not a simple extension of adult muscle biology but a reorganization of the tissue ecosystem [6, 7, 13, 14]. Myofibers, muscle stem cells, fibro‐adipogenic progenitors, immune cells, vascular cells, stromal populations, and neuromuscular components each acquire distinct aging‐associated states, while their communication networks become progressively distorted [6, 7]. In parallel, circulating proteomic and metabolomic studies suggest that muscle aging is shaped by systemic signals from adipose tissue, liver, gut, immune system, and nervous system. Together, these advances have shifted the field from a myofiber‐centered model toward a multiscale and interorgan framework [8, 11, 15].

These developments make it timely to reassess skeletal muscle aging through a broader framework that connects emerging mechanisms with therapeutic translation. Classical mechanisms such as impaired proteostasis, mitochondrial dysfunction, inflammation, fibrosis, and denervation remain central, but they are now increasingly interpreted within the context of niche remodeling, altered intercellular communication, systemic regulation, and functional decline [6, 11]. At the same time, therapeutic development is becoming more mechanism‐oriented, with growing interest in interventions targeting mitochondrial biology, myostatin/activin signaling, senescence, inflammation, extracellular vesicles, regenerative pathways, and neuromuscular maintenance. Yet translation remains constrained by biological heterogeneity, incomplete biomarkers, and insufficient endpoint alignment [11, 16]. Increasing lean mass alone may not restore mobility if muscle quality, neural control, metabolic function, and tissue repair remain impaired [4, 8, 11].

This review synthesizes recent progress in skeletal muscle aging from cellular mechanisms to therapeutic translation. Rather than presenting sarcopenia as a single‐pathway disorder, we organize the evidence around a multiscale model of muscle health. We first summarize the cellular and molecular landscape of aged skeletal muscle, emphasizing tissue‐level hallmarks, cellular ecosystem remodeling, and insights from single‐cell and multi‐omics studies. We then discuss core mechanisms driving muscle aging, including proteostasis imbalance, mitochondrial dysfunction, inflammaging, cellular senescence, stem cell exhaustion, extracellular matrix remodeling, fibro‐adipogenic conversion, and neuromuscular junction instability.

The review further considers systemic regulation and interorgan crosstalk, including endocrine and metabolic signals, communication with adipose tissue, liver, gut, and brain, and circulating mediators as biomarkers or therapeutic clues. Finally, we evaluate therapeutic interventions ranging from exercise and nutrition to pharmacological, regenerative, and advanced targeted strategies, with particular attention to the gap between preclinical promise and clinical benefit. By integrating recent mechanistic and translational evidence, this review aims to define skeletal muscle aging not merely as loss of mass, but as a multidimensional decline in tissue resilience, repair capacity, systemic coordination, and functional performance. This framework may help guide future work toward precision interventions that match biological mechanisms with appropriate patients, biomarkers, and clinically meaningful outcomes.

## 2 Cellular and Molecular Landscape of Skeletal Muscle Aging

Skeletal muscle aging is expressed at multiple levels, from visible tissue deterioration to subtle shifts in cellular states and communication networks. While the classical description of aging muscle has emphasized loss of mass and preferential atrophy of fast‐twitch fibers, recent work indicates that functional decline is more closely related to changes in muscle quality, tissue architecture, and microenvironmental coordination [4, 17]. Together, these changes provide the structural and cellular basis for understanding the mechanisms that drive age‐related functional decline.

### 2.1 Tissue‐Level Hallmarks of Skeletal Muscle Aging

At the tissue level, skeletal muscle aging is characterized by reduced fiber size, preferential vulnerability of fast‐twitch fibers, intramuscular fat accumulation, extracellular matrix (ECM) remodeling, fibrosis, vascular decline, neuromuscular deterioration, chronic low‐grade inflammation, and ionic dyshomeostasis [4, 11, 18, 19, 20]. These tissue‐level changes reflect the convergence of canonical aging processes, including loss of proteostasis, mitochondrial dysfunction, deregulated nutrient sensing, cellular senescence, stem cell exhaustion, epigenetic alterations, genomic instability, telomere attrition, and altered intercellular communication [4, 11, 18, 19, 20]. Their clinical relevance lies not only in muscle mass loss, but also in the progressive deterioration of muscle quality, contractile performance, metabolic flexibility, and repair capacity [4, 11]. Recent studies further indicate that functional decline is more closely associated with qualitative tissue remodeling than with absolute mass reduction alone [4, 6]. Among these changes, ECM reorganization, myosteatosis, chronic low‐grade inflammation, and senescence‐associated tissue reprogramming are particularly important because they reshape the aged muscle microenvironment [21, 22, 23, 24]. By disrupting mechanical integrity, metabolic homeostasis, vascular and neuromuscular support, and intercellular communication, these processes mark a shift from a regenerative tissue state toward a degenerative and poorly adaptive muscle niche [6, 23].

### 2.2 Cellular Ecosystem Remodeling in Aged Muscle

Skeletal muscle is a highly coordinated multicellular ecosystem composed not only of myofibers, but also of satellite cells, fibro‐adipogenic progenitors (FAPs), immune cells, vascular cells, Schwann cells, and other stromal populations [18]. Aging disrupts this ecosystem at multiple levels, including cell composition, functional state, and intercellular signaling [6, 18]. As a result, the aged muscle niche becomes less regenerative and more permissive to inflammation, fibrosis, denervation‐associated remodeling, and metabolic dysfunction. Recent human single‐cell and multimodal atlases have further shown that age‐related decline is not driven by a uniform program across all compartments, but instead reflects cell type‐specific vulnerabilities and maladaptive crosstalk within the tissue microenvironment [7].

#### 2.2.1 Myofibers

Myofibers remain the principal contractile units of skeletal muscle, but in aged muscle they undergo profound structural and metabolic deterioration. In addition to reduced fiber size, particularly in fast‐twitch fibers, aging myofibers display impaired mitochondrial function, altered excitation–contraction coupling, defective proteostasis, and increased susceptibility to denervation‐related stress [25, 26]. These changes compromise force generation and endurance even when gross muscle mass is relatively preserved. Importantly, recent multimodal studies suggest that myofiber aging should not be viewed in isolation, because fiber dysfunction is closely linked to changes in the surrounding niche, including inflammatory cues, ECM remodeling, and reduced trophic support from neural and vascular compartments. Thus, myofibers in aged muscle are both effectors and sensors of broader tissue ecosystem failure [17].

#### 2.2.2 Satellite Cells

Satellite cells are the canonical muscle stem cells (MuSCs) required for regeneration, yet their function declines markedly with age [27]. This decline is no longer understood simply as stem cell depletion. Instead, aged satellite cells exhibit impaired quiescence maintenance, delayed activation, defective self‐renewal, and reduced myogenic competence [28]. The recent human skeletal muscle aging atlas showed that distinct MuSC subsets display decreased ribosome biogenesis signatures and increased CCL2 expression, indicating that aging reshapes both their biosynthetic capacity and inflammatory phenotype [6, 29]. These findings support the idea that satellite cell dysfunction is partly niche‐driven: aged MuSCs exist within a microenvironment characterized by altered ECM, chronic inflammatory signaling, and disrupted cell–cell communication. As a result, their regenerative response becomes weaker and less coordinated, contributing to incomplete repair and progressive tissue degeneration.

#### 2.2.3 Fibro‐Adipogenic Progenitors and Stromal Cells

Among mononuclear cell populations, FAPs and related stromal cells have emerged as central regulators of the aged muscle niche [30, 31]. Under physiological conditions, FAPs support regeneration in a transient and tightly controlled manner; however, with aging they increasingly adopt maladaptive states linked to fibrosis, adipogenic conversion, and inflammatory amplification [30, 32]. Recent studies indicate that senescent or dysfunctional FAPs can recruit macrophages and reinforce inflammatory circuits through chemokine secretion, while broader stromal remodeling in aged human muscle is enriched for pro‐inflammatory and pro‐fibrotic pathways, including IL6/AP1 and TGFβ‐associated programs [33]. This makes FAPs particularly important not only as precursors of fibrotic and fatty infiltration, but also as niche integrators that translate extracellular and inflammatory signals into long‐lasting tissue remodeling. Accordingly, aging‐associated stromal dysregulation is now considered a major driver of microenvironmental decline rather than a secondary byproduct of myofiber loss.

#### 2.2.4 Immune, Vascular, and Neuromuscular Components

**FIGURE 1:**
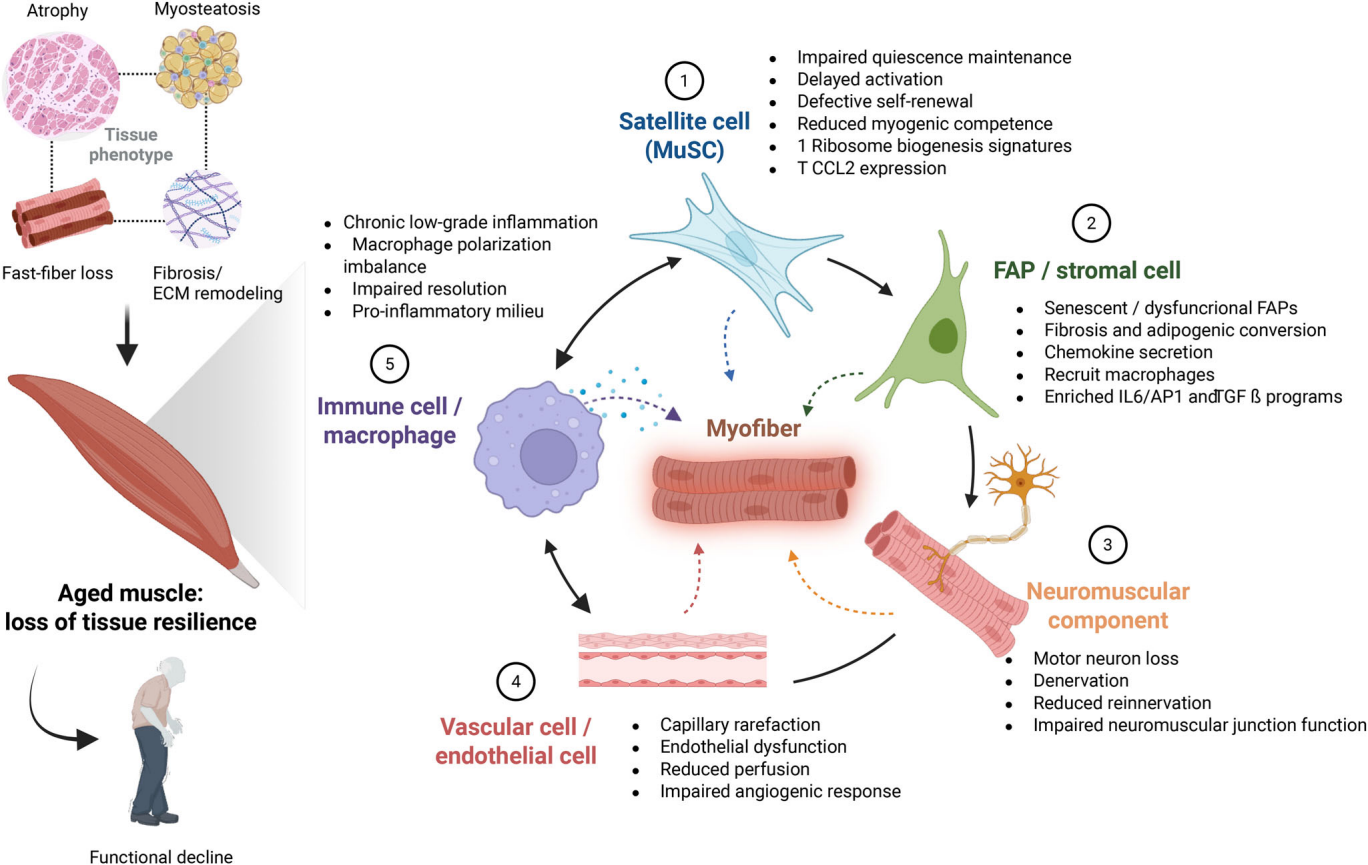
Cellular ecosystem remodeling and tissue‐level decline in aged skeletal muscle. Aged skeletal muscle shows tissue‐level deterioration, including fiber atrophy, fast‐fiber loss, myosteatosis, and fibrosis/extracellular matrix (ECM) remodeling, which together reduce tissue resilience and promote functional decline. This phenotype reflects coordinated remodeling of the muscle niche: muscle stem cells/satellite cells(MuSCs)lose regenerative competence; fibro‐adipogenic progenitors(FAPs)and stromal cells acquire fibrotic, adipogenic, and inflammatory states; immune cells sustain low‐grade inflammation and impaired resolution; vascular cells show reduced perfusion and angiogenic capacity; and neuromuscular components undergo denervation and impaired neuromuscular junction function. These interacting changes shift aged muscle from an adaptive, regenerative ecosystem toward a degenerative niche with reduced repair capacity and functional reserve. Figure created with BioRender.com.

Aged muscle also undergoes major alterations in nonmyogenic support systems. Immune remodeling is characterized by chronic low‐grade inflammation and altered cytokine and chemokine signaling, which can impair regeneration and sustain senescence‐associated tissue states [34]. In parallel, vascular decline reduces nutrient and oxygen delivery, weakens metabolic resilience, and limits efficient repair [35]. Neuromuscular changes, including motor unit instability, denervation, and neuromuscular junction deterioration, further exacerbate myofiber dysfunction and preferentially affect fast motor units [36]. Recent cell atlases emphasize that these compartments are not independent: inflammatory, vascular, glial, and stromal signals converge to shape the aged niche, while denervation‐related stress feeds back onto both myofibers and stem/progenitor cells [37]. Therefore, cellular ecosystem remodeling in aged muscle should be understood as a multicellular network failure, in which loss of regenerative coordination is as important as dysfunction in any single cell type (Figure 1).

### 2.3 New Insights From Recent Single‐Cell, Spatial, and Multi‐Omics Studies

Single‐cell, single‐nucleus, and multimodal profiling studies have reshaped the understanding of skeletal muscle aging [6, 7, 13, 38, 39] (Table 1). Rather than depicting aged muscle as a uniformly deteriorating tissue, these approaches reveal a highly heterogeneous process involving cell type‐specific vulnerabilities, compensatory responses, and altered intercellular communication. Human skeletal muscle aging atlases have identified distinct aging‐associated states in muscle stem cells, myofibers, stromal cells, immune populations, and neuromuscular compartments, demonstrating that regenerative decline emerges from coordinated dysfunction across the tissue ecosystem rather than from myofiber atrophy alone [6, 7, 14]. In particular, recent datasets show that aged MuSC subsets exhibit reduced ribosome biogenesis and enhanced inflammatory signaling, while stromal and microenvironmental populations display pro‐fibrotic and immune‐recruiting programs [6, 13, 40, 41]. Multimodal analyses integrating transcriptomic and epigenomic information further indicate that age‐related remodeling extends beyond gene expression changes to chromatin‐level reprogramming, thereby linking cellular state transitions to broader regulatory architecture [7, 13, 38, 39]. Spatial approaches add a further layer by showing where these altered cell states and signaling programs occur within tissue: spatial transcriptomic maps of mouse muscle regeneration across the lifespan identify senescent‐like MuSC and progenitor populations enriched in aged injury zones, while spatial and single‐cell analyses of elderly human muscle regeneration reveal FAP‐monocyte/macrophage colocalization and C3‐associated communication within regenerating regions [42, 43]. Human muscle aging resources further illustrate how spatial technologies can localize aging‐, frailty‐, and load‐responsive genes, including secreted or inflammatory mediators, to specific cell types and tissue regions [44].

**TABLE 1:** Recent single‐cell, single‐nucleus, spatial, and multi‐omics studies defining the cellular landscape of skeletal muscle aging.

Study	Species/sample	Omics strategy	Main cell types or compartments	Key aging‐related findings	Main contribution
Kedlian et al. [6]	Human skeletal muscle	Single‐cell/single‐nucleus transcriptomic atlas	Myofibers, MuSCs, FAPs, stromal, immune, vascular and neuromuscular‐associated populations	Cell type‐specific aging states; impaired MuSC biosynthesis and inflammatory shift	Human skeletal muscle aging atlas
Lai et al. [7]	Human skeletal muscle	Multimodal single‐cell atlas	Myonuclei, MuSCs, stromal, immune, endothelial and neuromuscular‐related compartments	Altered cell states, cell‐cell communication and chromatin regulation	Multimodal human aging muscle atlas
Burton et al. [12]	Myoblasts from older adults	Single‐cell RNA‐seq	Human myoblast populations	Myoblast heterogeneity linked to muscle mass and function	Connects human myogenic cell states with clinical phenotype
Li et al. [13]	Human skeletal muscle	Single‐nucleus multiomics plus senescence profiling	Myofibers, MuSCs, stromal, immune and senescent cell populations	Cell type‐specific senescence and SASP programs; CCR5/Maraviroc axis	Links muscle aging atlas to senotherapeutic discovery
Perez et al. [14]	Human skeletal muscle	Single‐nucleus RNA‐seq	Myonuclei and mononuclear cell populations	Aging‐, frailty‐ and senescence‐associated nuclear signatures	Early single‐nucleus map of aged human muscle
Moiseeva et al. [29]	Mouse skeletal muscle/regeneration model	Senescence atlas with single‐cell‐level niche analysis	Senescent cells, MuSCs, FAPs and immune/stromal populations	Aged‐like inflamed niche blunts muscle regeneration	Defines senescence‐associated regenerative niche failure
Varshney et al. [38]	Human skeletal muscle	Population‐scale single‐nucleus multi‐omics	Major myogenic and nonmyogenic compartments	Cell type‐specific regulatory programs and genetic regulation	Population‐scale single‐nucleus multi‐omic resource
Orchard et al. [39]	Human and rat skeletal muscle	Single‐nucleus multi‐omic integration	Myogenic and nonmyogenic nuclei across species	Regulatory elements and trait‐linked cell types across species	Cross‐species regulatory framework for skeletal muscle
Lazure et al. [40]	Skeletal muscle stem cell niche model	Transcriptomic profiling	MuSCs and niche‐associated regulatory inputs	Niche‐driven transcriptional reprogramming of MuSCs	Supports extrinsic regulation of MuSC aging
Benjamin et al. [41]	Skeletal muscle stem cells	Multi‐omics analysis	MuSCs/skeletal muscle stem cell states	Glutathione metabolism drives divergent stem cell aging states	Links metabolic heterogeneity to MuSC aging
Walter et al. [42]	Mouse skeletal muscle	Spatial and single‐cell transcriptomics	MuSCs, progenitors, immune cells, FAPs and endothelial cells	Senescent‐like MuSC/progenitor states enriched in aged injury zones	Spatiotemporal atlas of muscle regeneration across lifespan
Brorson et al. [43]	Human skeletal muscle	Spatial and single‐cell transcriptomics	FAPs, monocytes/macrophages and regenerating muscle regions	FAP‐macrophage colocalization and C3‐mediated communication	Defines spatial FAP‐macrophage crosstalk in elderly muscle repair
Stokes et al. [44]	Human skeletal muscle	Spatial and bulk transcriptomics	Myofibers, endothelial cells, fibroblasts and spatial muscle regions	Spatial localization of aging‐, frailty‐ and load‐responsive genes	Network‐based human muscle aging atlas with spatial validation

A central message from these studies is that skeletal muscle aging should be understood as a network‐level process. Altered ligand‐receptor interactions, distorted immune‐stromal signaling, disrupted myofiber‐stem cell communication, and impaired vascular or neuromuscular support all contribute to loss of tissue resilience. Thus, spatial omics does not replace single‐cell atlases but helps test whether inferred interactions actually occur within the same physical niche, such as inflammatory, fibrotic, or regenerating regions of aged muscle. This perspective provides a bridge from descriptive cellular atlases to mechanistic questions. If aged muscle is defined by altered cell states and disrupted niche coordination, the next step is to identify the molecular mechanisms that drive these changes and determine how they reinforce one another during functional decline.

## 3 Core Mechanisms Driving Skeletal Muscle Aging

The cellular and tissue‐level changes described above arise from interacting mechanisms that progressively weaken muscle maintenance, repair, and functional adaptation. Classical mechanisms such as impaired proteostasis, mitochondrial dysfunction, chronic inflammation, stem cell exhaustion, fibrosis, and denervation remain central to skeletal muscle aging [18]. However, they are best interpreted as connected processes rather than independent hallmarks. Defective protein quality control increases metabolic and organelle stress; mitochondrial dysfunction amplifies oxidative and inflammatory signaling; senescent and immune cells reshape the stromal niche; fibrosis and altered mechanics restrict regeneration; and neuromuscular instability drives myofiber remodeling and functional decline. Together, these interactions progressively weaken aged muscle's capacity to maintain homeostasis, repair damage, and recover from stress.

### 3.1 Proteostasis Imbalance and Anabolic Resistance

Skeletal muscle depends on continuous protein renewal to maintain contractile function, metabolic capacity, and repair competence [45]. With aging, this renewal becomes inefficient as anabolic signaling, proteolytic transcriptional control, autophagy‐lysosomal clearance, and myofibrillar remodeling become uncoupled. In this context, anabolic resistance involves two related but distinct defects. The first is impaired nutrient sensing, particularly reduced responsiveness to leucine and other essential amino acids, which weakens mTORC1 activation and downstream S6K1 signaling [46, 47, 48, 49]. This defect may involve amino acid‐sensing machinery upstream of mTORC1, including leucine‐ and arginine‐sensitive regulators such as Sestrin2 and CASTOR1 and their interaction with the GATOR complexes [50, 51]. The second is blunted endocrine and insulin‐like signaling through the IRS‐1/PI3K/Akt pathway, which limits the anabolic response to insulin, IGF‐1, nutrition, and mechanical loading [52]. Thus, anabolic resistance in aged muscle reflects a combined failure of amino acid sensing and growth factor signaling to coordinate protein synthesis with remodeling, rather than a simple reduction in postprandial protein synthesis. Reduced IGF‐1/PI3K/Akt activity also engages a direct catabolic transcriptional program. Under anabolic conditions, Akt phosphorylates FOXO1 and FOXO3a, restricting their nuclear activity [53]. When Akt signaling is insufficient, FOXO1/3a translocate to the nucleus and induce ubiquitin‐proteasome components, including the E3 ubiquitin ligases TRIM63/MuRF1 and FBXO32/MAFbx [52, 53]. This pathway links impaired anabolic signaling to active myofibrillar protein degradation. A second growth‐suppressive mechanism is the Myostatin/Activin A pathway. Myostatin and Activin A signal through activin type II receptors to promote Smad2/3 phosphorylation; activated Smad2/3 complexes then regulate transcriptional programs that oppose muscle growth [54]. Together, impaired IRS‐1/PI3K/Akt signaling, FOXO1/3a‐mediated proteolysis, and Myostatin/Activin A‐Smad2/3 signaling define proteostasis imbalance as an integrated signaling and transcriptional network rather than a descriptive phenotype. Yet anabolic resistance captures only part of the proteostatic defect [45]. Aged muscle may activate translation under certain conditions but still fail to rebuild mass and function, indicating that synthesis, degradation, folding, organelle clearance, and structural remodeling are no longer properly coordinated [45, 55]. This distinction is evident during recovery from disuse or injury [56, 57]. Old muscle can show increased translational capacity and myofibrillar protein synthesis during reloading, yet still display incomplete restoration of muscle mass and function [55, 56]. Such findings indicate that aged muscle is not limited only by insufficient protein synthesis [55]. Defective removal of damaged proteins, impaired autophagy‐lysosomal activity, inadequate remodeling of myofibrillar structures, and failure to reintegrate newly synthesized proteins into functional contractile architecture may all prevent synthetic activity from producing effective recovery [55, 58, 59]. This defective quality‐control response may involve disturbed ubiquitin‐proteasome proteolysis, impaired lysosomal function, and ER‐stress/CHOP activation rather than a simple failure to initiate translation [45, 55, 58, 59]. Autophagy and lysosomal quality control are therefore central to muscle aging [58, 60]. Basal autophagy removes damaged proteins and organelles, whereas impaired autophagy permits dysfunctional cellular components to accumulate [60, 61]. Chaperone‐mediated autophagy has gained particular relevance because it provides selective degradation of soluble proteins and appears to decline in aged skeletal muscle [62]. Loss of this pathway affects not only mature myofiber maintenance but also muscle stem cell fate, linking proteostasis to regeneration rather than to contractile tissue alone [63].Anabolic signaling remains modifiable, but its interpretation requires caution [49]. Exercise and amino acid‐based interventions can engage mTORC1‐related pathways in aged muscle, and nutritional formulations may improve fiber size, contractile indices, and anabolic signaling in old animals [64, 65]. However, mTOR activation alone is unlikely to restore aging muscle if FOXO‐driven proteolysis, Myostatin/Activin A‐Smad2/3 signaling, autophagy‐lysosome dysfunction, ER stress, and structural repair defects remain unresolved [45, 50, 53, 54, 65, 66]. The more relevant therapeutic goal is to restore proteostatic balance: synthesis must be coupled to degradation, folding, clearance, and tissue remodeling [45, 58, 67]. Thus, anabolic resistance should be integrated with amino acid sensing, IRS‐1/PI3K/Akt signaling, FOXO1/3a‐dependent transcription of TRIM63/MuRF1 and FBXO32/MAFbx, Myostatin/Activin A‐Smad2/3 signaling, mTORC1/S6K1 activity, autophagy‐lysosome function, chaperone‐mediated autophagy, ER‐stress control, and myofibrillar remodeling, rather than treated as an isolated defect in protein synthesis. In this sense, proteostasis imbalance provides an upstream entry point into mitochondrial dysfunction, inflammatory activation, and regenerative failure [45, 60, 61].

### 3.2 Mitochondrial Dysfunction and Impaired Quality Control

Mitochondrial dysfunction remains one of the most reproducible features of aged skeletal muscle, but its meaning has broadened beyond reduced oxidative capacity or increased reactive oxygen species [68]. The central issue is mitochondrial quality control: the ability to maintain a functional mitochondrial network through AMPK‐PGC‐1α‐linked biogenesis and metabolic adaptation, fusion–fission dynamics, mitophagy, and mitochondrial proteostasis [68, 69, 70]. When these processes become uncoupled, mtDNA mutation or deletion burden, impaired mitochondrial Ca^2^^+^ uptake, defective organelle turnover, and persistent redox stress contribute to energetic insufficiency, inflammatory signaling, and impaired contractile performance [71, 72, 73]. This broader interpretation is supported by single‐nucleus and multi‐omics analyses showing that aged myofibers acquire stress‐related, metabolic, denervation‐associated, and regeneration‐like transcriptional states [74, 75]. These findings place mitochondrial dysfunction within a wider remodeling of myofiber identity rather than as an isolated biochemical defect [73, 74]. Mitochondrial changes are therefore closely linked to excitation–contraction coupling, calcium handling, fatigue resistance, denervation‐responsive gene programs, and impaired adaptation to exercise or stress [72, 75, 76]. Mitophagy is a key part of this process [68, 77]. Damaged mitochondria must be recognized and removed before they become persistent sources of ROS, oxidized macromolecules, and inflammatory danger signals [77, 78].

At the molecular level, this process is mediated in part by the PINK1/Parkin pathway. Loss of mitochondrial membrane potential stabilizes PINK1 on the outer mitochondrial membrane, where it promotes recruitment and activation of the E3 ubiquitin ligase Parkin. Parkin then ubiquitinates outer mitochondrial membrane proteins, including VDAC1 and mitofusins such as Mfn1 and Mfn2, thereby marking damaged mitochondria for selective autophagic removal [79, 80]. These ubiquitin signals recruit mitophagy receptors, including OPTN and NDP52, which connect ubiquitinated mitochondria to LC3/GABARAP‐positive phagophores and promote mitochondrial engulfment by autophagosomes [81]. Thus, mitophagy is not only a macrolevel turnover pathway but also a spatially organized protein‐interaction cascade that couples mitochondrial damage sensing to autophagosome formation.

Experimental models of disuse indicate that intact mitophagy protects skeletal muscle from excessive atrophy, weakness, and mitochondrial remodeling [78]. This is especially relevant to aging because older adults are vulnerable to periods of inactivity, hospitalization, immobilization, and illness [78, 82]. When mitophagy is insufficient, transient catabolic stress may leave long‐lasting effects on muscle quality and function [77, 78]. Mitochondrial dynamics provide a second protein‐level quality‐control mechanism. Fusion maintains network connectivity and functional complementation, whereas fission helps segregate damaged mitochondrial segments for removal [68, 69, 70]. In aged or stressed myofibers, this balance may shift toward fragmentation. A central regulator of this transition is OPA1, an inner mitochondrial membrane GTPase that maintains inner membrane fusion and cristae organization. Stress‐activated OMA1 cleaves long OPA1 isoforms, reducing fusion‐competent OPA1 and increasing short OPA1 forms, thereby pushing the mitochondrial network toward fragmentation [83]. In skeletal muscle, age‐associated loss or impairment of OPA1 has been linked to mitochondrial dysfunction, muscle loss, systemic metabolic disturbance, and inflammatory remodeling [84]. Therefore, excessive OMA1‐dependent OPA1 processing provides a mechanistic link between mitochondrial stress and the fragmented mitochondrial architecture observed in aging muscle.

Mitochondrial dysfunction also reinforces proteostasis failure [68]. Damaged mitochondria generate oxidized proteins and lipids that burden the proteasome, autophagy, and chaperone systems [71, 73]. Conversely, impaired autophagy allows dysfunctional mitochondria to accumulate [77, 78]. This reciprocal relationship helps explain why mitochondrial and proteostatic defects often coexist in aged muscle [68, 69]. It also explains why interventions aimed only at antioxidant suppression may be less effective than strategies that improve mitochondrial turnover, AMPK‐regulated mitochondrial dynamics, metabolic flexibility, and stress adaptation [70, 85]. Functionally, mitochondrial impairment helps explain why muscle performance can deteriorate before severe atrophy is evident [76, 86, 87]. Reduced ATP availability, altered mitochondrial Ca^2^^+^ uptake, impaired excitation‐contraction coupling, increased mitochondrial fragmentation, and defective mitophagy can weaken muscle even when lean mass is relatively preserved [72, 76, 86]. Mitochondrial dysfunction therefore connects mtDNA damage, defective PINK1/Parkin‐mediated mitophagy, OMA1‐OPA1‐dependent network fragmentation, redox stress, and impaired bioenergetics to the clinical concept of muscle quality [76, 82, 87].

### 3.3 Inflammaging, Cellular Senescence, and Immune Dysregulation

Inflammation in aged muscle is better understood as a structured remodeling of the macrophage‐stromal‐myogenic niche than as a nonspecific rise in inflammatory markers [88, 89]. Earlier models focused mainly on elevated cytokines or macrophage infiltration [90, 91]. Current evidence points to a more complex disturbance involving macrophage immunometabolism, immune‐cell state transitions, timing, spatial organization, and communication with stromal and myogenic cells [89, 92, 93]. Aged muscle does not simply contain more inflammation; it loses the ability to mount, resolve, and coordinate immune responses in a regenerative sequence [89, 94, 95]. At the myofiber level, one key mediator of this inflammatory remodeling is the IL‐6/JAK/STAT3 pathway. Chronic IL‐6 exposure activates JAK‐dependent STAT3 phosphorylation, and sustained STAT3 signaling can promote muscle wasting while impairing anabolic and insulin‐responsive pathways [96, 97]. Mechanistically, STAT3 induces suppressor of cytokine signaling 3 (SOCS3), which interferes with insulin receptor substrate signaling by inhibiting IRS‐1 phosphorylation and weakening downstream PI3K/Akt activation in myofibers [97, 98]. This IL‐6/STAT3/SOCS3‐IRS‐1 axis provides a direct molecular link between inflammaging, insulin resistance, anabolic resistance, and proteolytic remodeling, and helps explain why chronic inflammation can accelerate muscle atrophy even without a primary defect in contractile proteins [98].

Cellular senescence is a major contributor to this altered niche [91, 99]. Senescent cells activate p16INK4a/p21CIP1‐associated programs and secrete senescence‐associated secretory phenotype (SASP) factors, including cytokines, chemokines, proteases, growth factors, and matrix‐remodeling molecules that influence neighboring cells [91, 100]. Among these SASP‐associated mediators, chemokine signaling is particularly important because it provides directional immune‐cell recruitment rather than a nonspecific inflammatory background. The CCL5/CCR5 axis is one such conduit: CCL5 acts as a chemotactic ligand for CCR5‐expressing monocytes/macrophages and other immune cells, thereby shaping macrophage accumulation and localization within damaged or chronically inflamed muscle [101]. Recent skeletal muscle work further suggests that enhanced CCL5 secretion can drive macrophage progenitor recruitment through CCR5‐dependent crosstalk and disturb FAP clearance, linking immune recruitment to stromal persistence, fibrotic remodeling, and impaired regeneration [102, 103]. Therefore, CCL5/CCR5 signaling should be viewed as a molecular bridge between macrophage chemotaxis and FAP‐centered niche remodeling, rather than as a generic inflammatory marker.

In skeletal muscle, senescence is particularly relevant when it occurs in niche‐forming populations such as fibro‐adipogenic progenitors, endothelial cells, immune cells, and muscle stem cells [99, 104, 105]. Senescent FAPs can recruit macrophages and modify macrophage behavior through their secretome, placing FAP senescence and SASP signaling upstream of immune remodeling rather than merely downstream of tissue damage [91, 104]. This FAP‐macrophage circuit may be reinforced by chemokine axes such as CCL5/CCR5 and by cytokine pathways such as IL‐6/STAT3/SOCS3, which together couple immune‐cell recruitment, myofiber insulin resistance, and stromal activation [103]. Mitochondrial stress may add another layer to this circuit, as damaged mitochondria can release mitochondrial DNA that activates cGAS‐STING signaling and strengthens sterile inflammatory and SASP‐related responses in aged muscle [106]. This stromal–immune interaction is central to the inflammatory aging phenotype [88, 89].

A further upstream trigger of sterile inflammation is mitochondrial stress. Damaged mitochondria and defective mitophagy can release mitochondrial DNA or other mitochondrial danger signals into the cytosol, where cGAS senses cytosolic DNA and activates STING‐dependent innate immune signaling [106]. cGAS‐STING activation induces type I interferon and NF‐κB‐related inflammatory programs, providing a mechanistic connection between mitochondrial dysfunction, cellular senescence, SASP amplification, and macrophage‐stromal remodeling [107].

Senescent stromal cells, altered macrophages, damaged myofibers, and dysfunctional mitochondria can form a self‐sustaining circuit [91, 100]. Inflammatory signals suppress anabolic responses, promote proteolysis, impair satellite cell differentiation, and bias FAPs toward fibrotic or senescent states [99, 104, 108]. At the same time, unresolved tissue damage and mitochondrial stress continue to feed inflammatory signaling [88, 89]. More specifically, IL‐6/STAT3/SOCS3 signaling can weaken IRS‐1/PI3K/Akt‐dependent anabolic responses in myofibers [96], CCL5/CCR5 signaling can sustain macrophage recruitment and FAP‐centered remodeling [101], and mtDNA/cGAS‐STING signaling can amplify sterile innate immune activation [106]. The result is not only chronic inflammation but a failure to convert inflammation into effective repair [89, 94, 95]. Regeneration studies further support this interpretation [94, 95, 109]. In young muscle, immune cells participate in a temporally ordered repair program: early inflammatory activity supports debris clearance, followed by a shift toward pro‐regenerative signaling [92, 94]. In aged muscle, this sequence is disrupted, with impaired inflammatory resolution, reduced IL‐33‐dependent Treg accumulation, and altered macrophage‐derived repair signals [89, 95]. Myogenic and nonmyogenic cells fail to coordinate properly, including Sepp1‐, GPNMB‐, and HGF/MET‐related macrophage programs, delaying repair and promoting persistent inflammatory and fibrotic remodeling [89, 93, 95]. This explains why broad anti‐inflammatory suppression may be insufficient or even counterproductive if it interferes with necessary repair signals [90, 94, 110]. Therapeutically, the more relevant goal is not simply to reduce inflammation, but to restore immune resolution and suppress pathological senescent signaling [91, 100, 110]. Targeting defined molecular conduits, such as IL‐6/STAT3/SOCS3, CCL5/CCR5, and cGAS‐STING signaling, together with senolytics, senomorphics, macrophage‐state modulation, and niche‐targeted approaches, may therefore be more informative than nonspecific cytokine blockade, provided that protective inflammatory responses required for regeneration are preserved.

### 3.4 Stem Cell Exhaustion and Regenerative Failure

Satellite cells are essential for skeletal muscle repair, but their aging phenotype cannot be reduced to numerical depletion [111, 112]. In many aged muscles, satellite cells remain present yet show impaired quiescence maintenance, delayed activation, defective self‐renewal, reduced differentiation capacity, and diminished stress tolerance [113, 114, 115]. The dominant problem is therefore loss of regenerative competence rather than simple absence of stem cells [111, 116]. Intrinsic defects contribute to this decline [112]. Aged satellite cells display altered metabolic control, mitochondrial stress, DNA damage responses, DNA methylation drift, impaired polarity, excessive p38MAPK and Wnt activity, reduced Notch‐p53 signaling, and JAK‐STAT/mTOR dysregulation [114, 117, 118, 119, 120]. Among these intrinsic defects, hyperactivation of p38α MAPK is a central molecular driver of aged MuSC exhaustion. In aged satellite cells, elevated FGF receptor‐1 and p38α/β MAPK activity impairs self‐renewal and disrupts the balance between symmetric stem‐cell‐expanding divisions and asymmetric divisions that generate committed progeny [114]. Excessive p38α MAPK activity weakens the establishment of stem‐cell polarity, biases aged MuSCs toward asymmetric fate segregation and premature myogenic commitment, and progressively depletes the quiescent self‐renewing pool [114, 121]. Pharmacological inhibition of p38α/β MAPK can partially restore self‐renewal capacity in aged satellite cells, supporting a causal role for this pathway rather than a merely correlative association [114, 121].

Age‐related dysregulation of Sprouty1 (Spry1) provides a second mechanism linking niche signals to quiescence failure. Spry1 is a receptor tyrosine kinase signaling antagonist that restrains FGF signaling in quiescent Pax7+ satellite cells [122, 123]. During regeneration, Spry1 is normally downregulated during activation and re‐induced as self‐renewing cells return to quiescence; loss of Spry1 prevents efficient re‐entry into quiescence and reduces the homeostatic satellite cell pool [122]. In aged muscle, increased niche‐derived FGF activity, together with insufficient Spry1‐mediated negative feedback, can drive inappropriate satellite cell activation under homeostatic conditions, resulting in loss of quiescence, stem cell depletion, and diminished regenerative capacity [123, 124]. Conversely, reducing FGFR1 signaling or restoring Spry1 expression protects aged satellite cells from depletion, highlighting the Spry1‐FGF axis as a core regulator of MuSC quiescence [123].

Metabolic regulation has become particularly important [125]. xCT/SLC7A11‐dependent cystine uptake, glutathione redox balance, and bioenergetic control can shape stem cell function, suggesting that metabolic resilience is part of the regenerative machinery rather than a secondary feature of aging [125, 126]. Protein quality control also influences satellite cell fate [112]. Decline in chaperone‐mediated autophagy and other proteostatic systems can compromise the ability of satellite cells to maintain quiescence, respond to activation cues, and complete myogenic differentiation [125]. A stem cell that cannot preserve proteome integrity during quiescence or activation may fail to regenerate tissue even if it remains numerically detectable [111, 116]. Extrinsic niche signals are equally decisive [112, 127]. Aged satellite cells reside in an environment characterized by altered ECM stiffness, chronic inflammatory cues, vascular dysfunction, denervation‐associated signals, and activated FAPs [112, 127, 128]. These extrinsic constraints can suppress regenerative potential even when some intrinsic stem cell function remains [115, 127]. Cytokine programs, including CCL2, CXCL8, IL‐6, TNFRSF12A, CSF‐1, and FGF2, regulate human satellite cell activation, whereas aged MuSC‐derived IL‐6/Spp1 signaling can drive mesenchymal progenitor expansion and fibrosis [113, 128]. FGF2 is particularly relevant because excessive niche‐derived FGF signaling can push aged satellite cells out of quiescence when Spry1‐mediated feedback is insufficient [114]. Microbiota‐derived systemic cues may further shape inflammatory tone and regenerative capacity [129, 130]. Thus, regenerative failure in aged muscle is best viewed as niche‐dependent stem cell dysfunction [112, 127]. Satellite cells are responsive units embedded in a tissue environment that can either support or restrict repair [127]. This distinction matters therapeutically [119, 128]. If aged satellite cells are present but suppressed by the niche, interventions that improve Notch/PGE2 signaling, limit p38α/β MAPK hyperactivation, restore Spry1‐FGF feedback, reduce JAK‐STAT or IL‐6/Spp1‐driven niche dysfunction, and restore ECM, immune, vascular, metabolic, or FAP support may partially restore regeneration without direct stem cell replacement [116, 119, 128]. If the regenerative pool is irreversibly depleted or deeply dysfunctional, more advanced regenerative strategies may be required.

### 3.5 Extracellular Matrix Remodeling, Fibrosis, and Fibro‐Adipogenic Conversion

The extracellular matrix has moved from the background of muscle aging to a central regulatory compartment [131, 132]. It is not merely a scaffold that accumulates collagen with age. It regulates force transmission, mechanical signaling, growth factor availability, immune cell trafficking, satellite cell behavior, and FAP fate [133, 134]. Changes in collagen alignment, cross‐linking, matrix composition, and stiffness can therefore alter muscle function even before large changes in muscle mass occur [133]. FAPs provide the main cellular link between ECM remodeling, fibrosis, fat infiltration, and inflammatory niche signaling [135, 136, 137]. In healthy repair, FAPs expand transiently and support myogenesis through type2 innate immune signals and trophic‐structural cues [138]. In aged or chronically stressed muscle, they can adopt fibrotic, adipogenic, inflammatory, or senescent states [139, 140]. This fibro‐adipogenic conversion is driven by defined signaling axes rather than by passive stromal drift. Canonical Wnt/β‐catenin signaling is one such axis. In FAPs and muscle‐resident mesenchymal cells, enhanced Wnt/GSK3β/β‐catenin activity can alter adipogenic–fibrogenic fate decisions, promote stromal expansion, and favor extracellular matrix‐producing programs [141, 142]. β‐catenin stabilization and nuclear transcriptional activity therefore provide a mechanism by which aged or chronically injured muscle shifts FAP behavior toward a fibrogenic niche state [141, 142].

A second major driver is TGF‐β/Smad3 signaling. TGF‐β1 activates TGF‐β receptors and induces Smad2/3 phosphorylation, with Smad3 acting as a canonical profibrotic transcriptional mediator. In FAPs, this pathway promotes myofibroblast‐like differentiation and the expression of extracellular matrix components, including collagen‐associated fibrotic programs [142]. Persistent TGF‐β/Smad3 activity therefore converts the normally transient reparative FAP response into sustained matrix deposition and fibrotic remodeling [142].

These pathways may be amplified by age‐related loss of antiaging systemic factors such as Klotho. Klotho has been linked to skeletal muscle regeneration and can antagonize excessive Wnt signaling in aged muscle stem cell contexts [143]. More broadly, Klotho can inhibit Wnt and TGF‐β signaling, suggesting that declining circulating or tissue Klotho with age may lower the threshold for Wnt/β‐catenin‐ and TGF‐β/Smad3‐driven fibrogenic remodeling [144]. Thus, Klotho loss provides a plausible upstream connection between systemic aging and local FAP‐centered fibrosis.

Fibrosis is therefore not simply excess matrix deposition after damage; it reflects an active stromal cell‐state program involving FAP‐macrophage coordination, Wnt/β‐catenin activation, TGF‐β/Smad3 signaling, mechanotransduction, and C1q‐sensitive fibrogenic signaling [43, 145]. Senescent and dysfunctional FAPs are especially important because they connect fibrosis, inflammation, and impaired regeneration [140, 145]. Their secretome can recruit and shape macrophages, while macrophage‐derived signals can further influence FAP behavior [146, 147]. FAP‐derived miR‐125b‐5p‐rich exosomes can promote pro‐regenerative macrophage polarization, whereas macrophage‐derived exosomes can regulate FAP adipogenesis [146, 147]. This stromal‐immune loop provides a mechanism by which a transient repair response becomes a chronic degenerative niche. It also helps explain why fibrosis, myosteatosis, and inflammation often coexist in aged muscle rather than appearing as separate pathologies [136]. Fibrosis impairs function through multiple routes [131, 132]. Increased matrix stiffness reduces tissue compliance and affects lateral force transmission [133]. Importantly, increased ECM stiffness is not only a mechanical consequence of fibrosis but also a feed‐forward signaling input. Stiff or aged muscle matrix can promote nuclear YAP/TAZ activation in fibroblasts and FAPs, inducing matricellular and myofibroblast‐like programs that further increase extracellular matrix deposition [132]. In FAPs, fibrotic matrix stiffness can activate YAP signaling and drive myofibroblast conversion, whereas soft matrices or inhibition of YAP/TAZ activity reduce TGF‐β‐dependent FAP differentiation into myofibroblasts [148]. This places YAP/TAZ mechanotransduction downstream of ECM stiffness and functionally linked to TGF‐β/Smad3 signaling in FAP pathology [142]. Abnormal ECM composition interferes with satellite cell activation and differentiation, including fibronectin loss and Tenascin‐C‐Annexin A2‐related niche signaling [134, 149]. Excessive matrix deposition can limit diffusion and vascular support. Fibro‐adipogenic conversion contributes to myosteatosis, which is associated with reduced muscle quality, metabolic dysfunction, and local inflammation [136, 137, 150]. These effects explain why individuals with similar muscle mass can differ markedly in strength and mobility. The key unresolved question is no longer whether ECM remodeling occurs, but which matrix programs are adaptive, which are maladaptive, and when they become irreversible [43, 145]. Matrix deposition is required for repair, whereas persistent fibrosis restricts repair [135, 138]. Future studies will need to resolve FAP subtypes, ECM ligands, collagen mechanics, Wnt/β‐catenin‐ and TGF‐β/Smad3‐dependent fibrogenic programs, Klotho‐sensitive antifibrotic regulation, YAP/TAZ‐mediated mechanotransduction, Musclin‐FILIP1L signaling, C1q‐responsive fibrosis, and immune interactions in human aging tissues [43, 145, 151]. Without this resolution, antifibrotic strategies risk suppressing repair as well as pathology.

### 3.6 Neuromuscular Junction Instability and Denervation‐Related Remodeling

Neuromuscular junction instability helps explain the gap between muscle mass and muscle strength [152, 153]. The neuromuscular junction (NMJ) converts motor neuron activity into muscle contraction; when this interface deteriorates, myofibers receive insufficient or irregular neural input, leading to impaired neuromuscular transmission, motor unit remodeling, altered fiber identity, and atrophy [154, 155, 156]. For this reason, denervation should not be treated only as a late downstream feature of sarcopenia. It can actively shape the aging muscle phenotype [157, 158]. Human studies support the clinical relevance of this mechanism [152]. Neuromuscular impairment differs across stages of sarcopenia, with evidence of altered motor unit function and NMJ instability. Circulating markers related to NMJ breakdown, such as C‐terminal agrin fragment, have also been explored in relation to muscle performance [159, 160, 161]. Mechanistically, CAF is generated by neurotrypsin‐dependent cleavage of agrin, a heparan sulfate proteoglycan required for NMJ formation and maintenance [162]. Neurotrypsin cleaves agrin at defined synaptic sites and releases C‐terminal agrin fragments, including the 22‐kDa CAF22 fragment, thereby providing a protease‐dependent molecular readout of agrin remodeling at the NMJ [162]. Because CAF reflects neurotrypsin‐mediated agrin cleavage and NMJ remodeling, it is best interpreted as a biomarker rather than a causal pathway [159, 160, 161, 162, 163, 164]. These findings do not yet provide a complete causal map, but they support the need to incorporate neural input into models of muscle aging [152, 163]. Animal and mechanistic studies further show that NMJ degeneration can appear early enough to contribute to functional decline [153]. Sarcopenic models exhibit acetylcholine receptor fragmentation, NMJ discontinuity, and altered Dok7‐related signaling [157]. Low‐magnitude high‐frequency vibration may preserve NMJ morphology by increasing Dok7 expression and suppressing ERK1/2 phosphorylation, whereas skeletal muscle agrin deficiency accelerates sarcopenia‐like decline [157, 158]. Mechanical stimulation or modulation of agrin‐associated pathways can improve aspects of NMJ morphology and function, suggesting that maintaining synaptic integrity may be necessary for preserving muscle performance [157]. Denervation also leaves a molecular imprint within myofibers [154, 165]. Sirt6‐dependent mono‐ADP‐ribosylation of YY1 promotes dystrophin expression and supports neuromuscular transmission, linking myofiber‐intrinsic epigenetic regulation to NMJ maintenance [154, 165]. Muscle reprogramming can also enhance reinnervation after peripheral nerve injury, indicating that the denervated myofiber is an active determinant of synaptic recovery [166]. This indicates that NMJ instability is not only a synaptic structural problem; it reshapes myofiber metabolism, contractile properties, stress responses, and inflammatory signaling [167]. Loss of neural input can promote fiber‐type grouping, mitochondrial dysfunction, proteolysis, and atrophy [154, 155]. Conversely, fibrosis, vascular dysfunction, inflammation, and altered muscle‐derived signals can impair NMJ maintenance. A key implication is that increasing muscle mass alone may not restore function if neural control remains unstable [152, 153]. Anabolic or anticatabolic strategies may therefore need to be combined with exercise, mechanical stimulation, motor unit preservation, or approaches that support Dok7/agrin‐associated synaptic stability, limit maladaptive neurotrypsin‐dependent agrin cleavage, dystrophin‐dependent neuromuscular transmission, and denervation‐responsive myofiber programs [154, 157, 158, 163, 164, 165].

**FIGURE 2:**
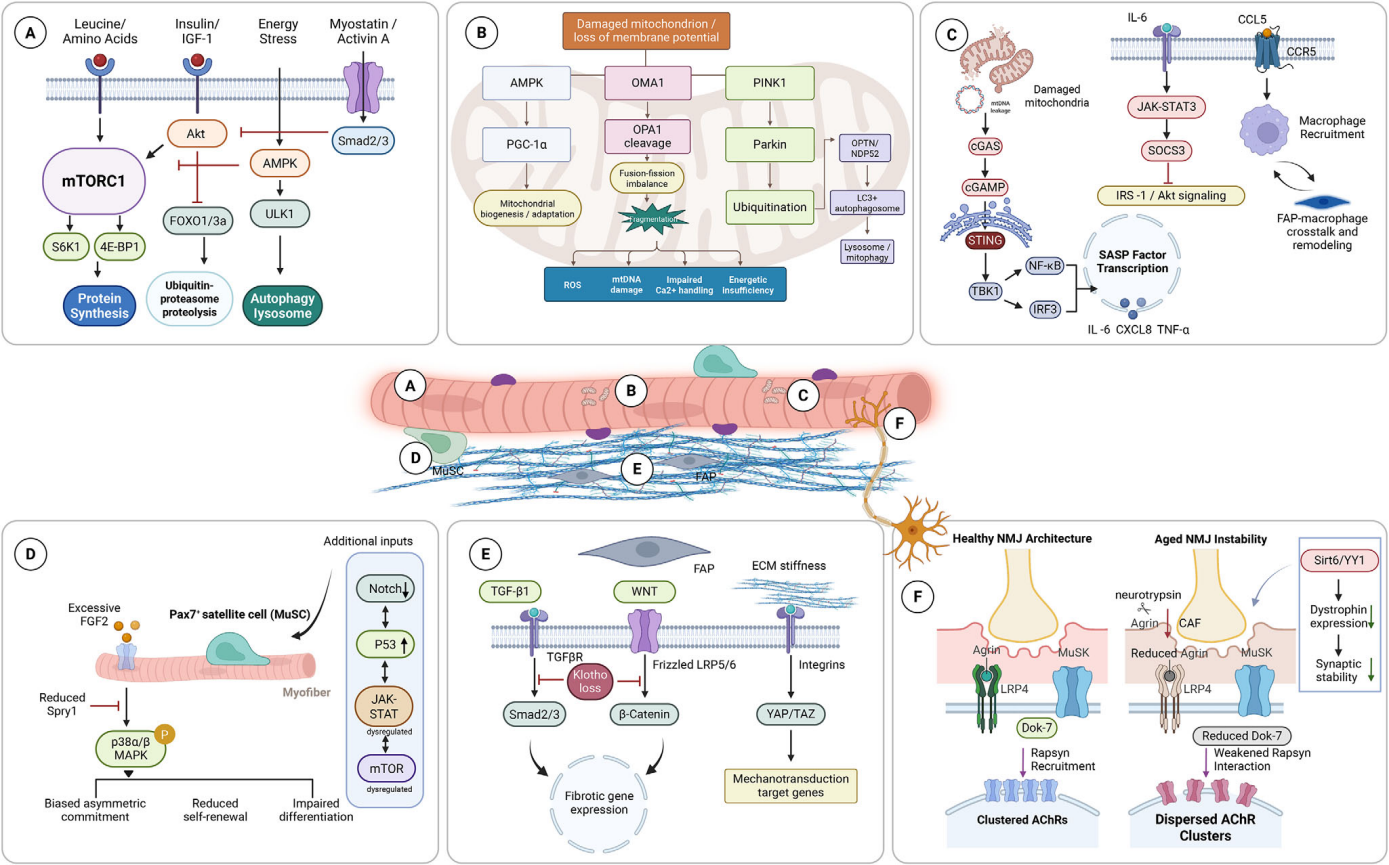
Core molecular mechanisms driving skeletal muscle aging. Schematic overview of six interconnected molecular mechanisms that progressively impair skeletal muscle homeostasis, regeneration, and function. (A) Proteostasis imbalance and anabolic resistance. Reduced responsiveness to leucine/amino acids and insulin/IGF‐1 weakens Akt‐mTORC1 signaling and its downstream effectors S6K1 and 4E‐BP1, thereby limiting protein synthesis. Reduced Akt activity also promotes FOXO1/3a‐dependent ubiquitin‐proteasome proteolysis, whereas energy stress activates the AMPK‐ULK1 axis to regulate autophagy‐lysosome activity. Myostatin/Activin A‐Smad2/3 signaling further suppresses muscle growth. (B) Mitochondrial dysfunction and impaired quality control. Loss of mitochondrial membrane potential activates PINK1‐Parkin‐dependent ubiquitination and recruitment of OPTN/NDP52 to LC3‐positive autophagosomes for mitophagy. Impaired AMPK‐PGC‐1α‐mediated mitochondrial biogenesis and excessive OMA1‐dependent OPA1 cleavage disrupt mitochondrial dynamics, promoting fragmentation, ROS accumulation, mtDNA damage, defective Ca^2^^+^ handling, and energetic insufficiency. (C) Inflammaging, cellular senescence, and immune dysregulation. Cytosolic mtDNA released from damaged mitochondria activates the cGAS‐cGAMP‐STING‐TBK1 pathway, leading to IRF3 and NF‐κB activation and transcription of SASP and inflammatory factors, including IL‐6, CXCL8, and TNF‐α. Chronic IL‐6‐JAK‐STAT3‐SOCS3 signaling suppresses IRS‐1/Akt activity, while CCL5‐CCR5 signaling promotes macrophage recruitment and FAP‐macrophage crosstalk. (D) Stem cell exhaustion and regenerative failure. Excessive FGF2 signaling together with reduced Spry1 feedback activates p38α/β MAPK in Pax7‐positive satellite cells, increasing asymmetric commitment while reducing self‐renewal and differentiation. Dysregulated Notch, p53, JAK‐STAT, and mTOR signaling further compromises regenerative competence. (E) ECM remodeling, fibrosis, and fibro‐adipogenic conversion. TGF‐β1‐TGFβR‐Smad2/3, Wnt‐Frizzled/LRP5/6‐β‐catenin, and ECM stiffness‐integrin‐YAP/TAZ pathways converge in FAPs to induce fibrotic and mechanotransduction‐related gene programs; reduced Klotho‐mediated restraint further facilitates maladaptive matrix remodeling. (F) NMJ instability and denervation‐related remodeling. In the healthy neuromuscular junction, Agrin activates the LRP4–MuSK complex, promoting Dok‐7 phosphorylation, rapsyn recruitment, and stable clustering of acetylcholine receptors. With aging, reduced Agrin signaling, decreased Dok‐7 activity, and weakened rapsyn interaction disperse AChR clusters and destabilize neuromuscular transmission. The Sirt6‐YY1‐dystrophin axis provides additional myofiber‐intrinsic support for synaptic stability. The central schematic indicates the anatomical localization of these mechanisms within the myofiber, satellite‐cell niche, extracellular matrix, stromal compartment, mitochondria, and neuromuscular junction. Figure created with BioRender.com.

In summary, skeletal muscle aging is driven by interacting mechanisms that converge on loss of tissue resilience [152, 158]. Proteostasis imbalance limits renewal of the contractile and regulatory proteome; mitochondrial dysfunction amplifies energetic and inflammatory stress; senescence and immune dysregulation reshape the stromal niche; satellite cell dysfunction compromises repair; ECM remodeling and FAP conversion alter mechanics and tissue composition; and NMJ instability weakens neural control while driving myofiber remodeling [166, 167] (Figure 2). These mechanisms are not parallel tracks. They reinforce one another through altered cell–cell communication, tissue mechanics, metabolic stress, and impaired repair. This local framework should also be placed within a broader systemic context, in which endocrine regulation, interorgan crosstalk, and circulating mediators shape muscle decline and therapeutic responsiveness.

## 4 Systemic Regulation and Interorgan Crosstalk in Muscle Aging

The mechanisms described above operate within skeletal muscle, but their trajectory is shaped by systemic endocrine, metabolic, inflammatory, microbial, mitochondrial, and neural signals [168, 169, 170]. Exercise‐induced exerkines and myokines, including IL‐6, irisin/FNDC5, apelin, brain‐derived neurotrophic factor (BDNF), myostatin‐related ligands, and extracellular vesicle cargoes, provide one route through which contracting muscle communicates with adipose tissue, liver, brain, immune cells, and vasculature [168, 169, 171]. Conversely, aging muscle is exposed to systemic inputs such as insulin resistance, altered lipid and amino acid metabolites, adipokines, hepatokines, microbial metabolites, and mitokines such as FGF21 and GDF15 [168, 170, 171, 172]. These signals can modify anabolic sensitivity, mitochondrial quality control, immune tone, regenerative capacity, fibrosis, and neuromuscular stability. Thus, interorgan crosstalk is not an abstract systemic background; it consists of identifiable mediators that can amplify or buffer local aging mechanisms. This perspective helps explain clinical heterogeneity. Similar weakness or mobility limitation may arise from different upstream combinations of insulin resistance, lipid toxicity, adipose inflammation, hepatic FGF21‐related stress, altered bile acid or amino acid metabolism, reduced microbial butyrate production, impaired exercise‐induced exerkine responses, or neurobehavioral decline [168, 170, 173, 174, 175, 176]. Systemic regulation therefore links local muscle biology to patient‐level differences in progression and treatment response.

### 4.1 Endocrine and Metabolic Regulation

Endocrine and metabolic changes provide major systemic constraints on aged muscle. Insulin resistance is closely linked to sarcopenia because impaired glucose disposal, reduced anabolic responsiveness, and lipid metabolic stress can converge on muscle protein turnover, mitochondrial function, and physical performance [172, 176, 177]. Myostatin and activin A represent catabolic endocrine signals with clinical relevance to sarcopenia, while apolipoprotein J and related circulating proteins may help define metabolic or inflammatory contexts associated with low muscle mass and function [178, 179, 180]. These factors should be interpreted cautiously: some are candidate biomarkers, whereas others are more directly involved in anabolic–catabolic regulation.

Metabolomic and lipidomic studies provide more specific molecular clues. Recent studies have associated sarcopenia or low muscle function with changes in lipid‐related metabolites, amino acid metabolites, bile acids, and carnitine‐related species [173, 174, 175, 181, 182]. These signatures suggest disturbances in fatty acid handling, mitochondrial substrate use, amino acid availability, and enterohepatic metabolism rather than a simple deficit in protein intake. Amino acid metabolites are relevant to mTORC1‐linked anabolic signaling; carnitine‐related metabolites reflect aspects of fatty acid transport and mitochondrial oxidation; and bile acids may function as systemic metabolic signals in addition to their digestive roles [174, 182, 183].

Exercise‐responsive metabolomics adds a dynamic dimension. Older adults can still mount circulating metabolic responses to acute resistance exercise, but several metabolites show age‐dependent patterns [184]. This suggests that systemic metabolism influences not only baseline muscle status, but also responsiveness to mechanical stimulation. Circulating metabolic profiles may therefore help identify whether muscle decline is accompanied by insulin resistance, lipid overload, amino acid imbalance, mitochondrial substrate stress, or impaired exercise responsiveness [173, 174, 175, 176, 177, 181, 182, 184].

### 4.2 Muscle Crosstalk With Adipose Tissue, Liver, Gut, and Brain

As an active endocrine tissue, skeletal muscle also releases myokines that help coordinate metabolism, inflammation, and communication with organs such as adipose tissue, liver, gut, and brain [185]. Adipose tissue affects muscle through lipid flux, adipokines, inflammatory mediators, and possibly extracellular vesicle‐related signaling [186, 187, 188]. In sarcopenic obesity, adipose dysfunction and intramuscular fat accumulation are linked to inflammation, oxidative stress, insulin resistance, mitochondrial dysfunction, and reduced muscle quality [186]. Clinical data also show that increased adipokines are associated with sarcopenia in nonobese women with rheumatoid arthritis, suggesting that adipose‐related inflammatory signaling can be relevant even outside obesity [187]. Conversely, reduced muscle mass and activity weaken glucose and lipid disposal, reinforcing adipose dysfunction [186, 188]. This creates a reciprocal muscle‐adipose loop in which metabolic disease and muscle decline may accelerate one another.

The liver provides another major systemic interface. Through glucose production, amino acid metabolism, lipid handling, bile acid signaling, ammonia detoxification, and hepatokine secretion, the liver can influence muscle metabolism and repair [189, 190, 191, 192]. Mechanistic studies make this axis more concrete. In nonalcoholic steatohepatitis, skeletal muscle IRF4‐regulated FSTL1 acts through hepatic DIP2A/CD14 signaling, identifying an IRF4‐FSTL1‐DIP2A/CD14 pathway linking muscle to liver pathology [189]. In the opposite direction, hepatocyte‐derived FGF21 mediates liver‐muscle crosstalk in decompensated cirrhosis and impairs muscle regeneration by inhibiting satellite cell myogenesis through KLB/β‐klotho‐associated PI3K/Akt signaling [190]. At the receptor level, endocrine FGF21 requires β‐Klotho (KLB) as an obligate co‐receptor and signals through FGFR complexes, particularly FGFR1c/β‐Klotho, to activate downstream MAPK/ERK1/2 and AMPK‐linked metabolic networks in target tissues [193]. In skeletal muscle, this receptor‐level mechanism helps distinguish FGF21 signaling from a nonspecific marker of hepatic or mitochondrial stress. FGF21 is also discussed as a hepatokine involved in metabolic dysfunction‐associated steatotic liver disease (MASLD) progression [191]. These findings indicate that liver‐muscle communication involves defined endocrine factors, not only nonspecific nutrient imbalance.

The gut‐muscle axis has become more mechanistically defined through microbiome and metabolome studies [194, 195, 196]. Gut microbiota can influence nutrient extraction, systemic inflammation, bile acid metabolism, amino acid availability, and short‐chain fatty acid production [194, 196]. Butyrate is particularly relevant because it acts as a histone deacetylase inhibitor and has been shown to improve metabolism and reduce age‐related muscle atrophy in experimental models [195]. Mechanistically, butyrate inhibits zinc‐dependent HDACs, with class I HDAC inhibition strongly linked to increased PGC‐1α expression, mitochondrial biogenesis, and oxidative metabolism in skeletal muscle [195, 197]. By increasing histone acetylation, butyrate can promote a more transcriptionally permissive chromatin state at metabolic gene regulatory regions, thereby favoring oxidative metabolism programs, including PGC‐1α‐centered mitochondrial gene networks [198]. Integrated gut microbiome and serum metabolome analyses further associate microbial and metabolic alterations with low handgrip strength in older adults [194]. Thus, microbial metabolites, bile acid profiles, and amino acid‐related signatures may connect gut ecology to muscle metabolism and function [174, 194, 195, 196].

Brain‐muscle communication adds a neural and behavioral layer. Motor neuron integrity, central drive, cognition, sleep, appetite regulation, motivation, and physical activity all influence muscle maintenance [199, 200]. Studies suggest that muscle‐derived mediators may participate in the relationship between sarcopenia and cognitive decline, whereas large‐scale UK Biobank data show associations between sarcopenic traits, brain structure, and cognitive performance [199, 200]. This axis is not mediated only by circulating molecules. Motor unit recruitment, physical activity behavior, mobility restriction, and feedback from functional decline to cognition and resilience are also central components.

### 4.3 Circulating Mediators as Biomarkers and Therapeutic Clues

Circulating mediators offer a practical window into these organ interactions. Candidate markers include proteins, metabolites, lipids, hormones, inflammatory mediators, extracellular vesicles, ECM fragments, microbial metabolites, and organ‐derived factors such as myokines, adipokines, hepatokines, and mitokines [201, 202, 203, 204]. Proteomic, metabolomic, lipidomic, and extracellular vesicle studies have identified signatures associated with sarcopenia, frailty, low muscle mass, weak grip strength, and poor physical performance [183, 201, 202, 203, 204, 205, 206].

Several examples illustrate why specific mediators are more useful than broad categories. Plasma proteomic studies have identified candidate proteins associated with age‐related sarcopenia and frailty [201, 205]. Extracellular vesicle proteomics has identified PF4 and C1R as potential biomarkers of sarcopenia, and other EV studies link vesicular cargoes to lower muscle mass, muscle function, and physical performance [202, 203]. Metabolomic studies highlight amino acid metabolites, bile acids, and carnitine‐related metabolites as candidate indicators of altered muscle metabolism [174, 182, 183, 206]. Endocrine studies suggest that myostatin, activin A, and apolipoprotein J may help characterize catabolic or metabolic‐inflammatory contexts associated with sarcopenia [178, 179, 180].

However, circulating mediators require cautious interpretation. Most are not muscle‐specific. FGF21 may reflect hepatic or mitochondrial stress, adipokines may reflect adipose or inflammatory disease activity, myostatin may indicate catabolic signaling, and EV proteins may derive from multiple tissues [178, 190, 201, 202, 203, 204]. Cross‐sectional associations also cannot establish whether a mediator causes muscle decline, compensates for tissue damage, or reflects comorbidity. Their strongest value is therefore not as single universal diagnostic markers, but as components of integrated profiles combining blood markers with imaging, functional testing, and clinical context.

**FIGURE 3:**
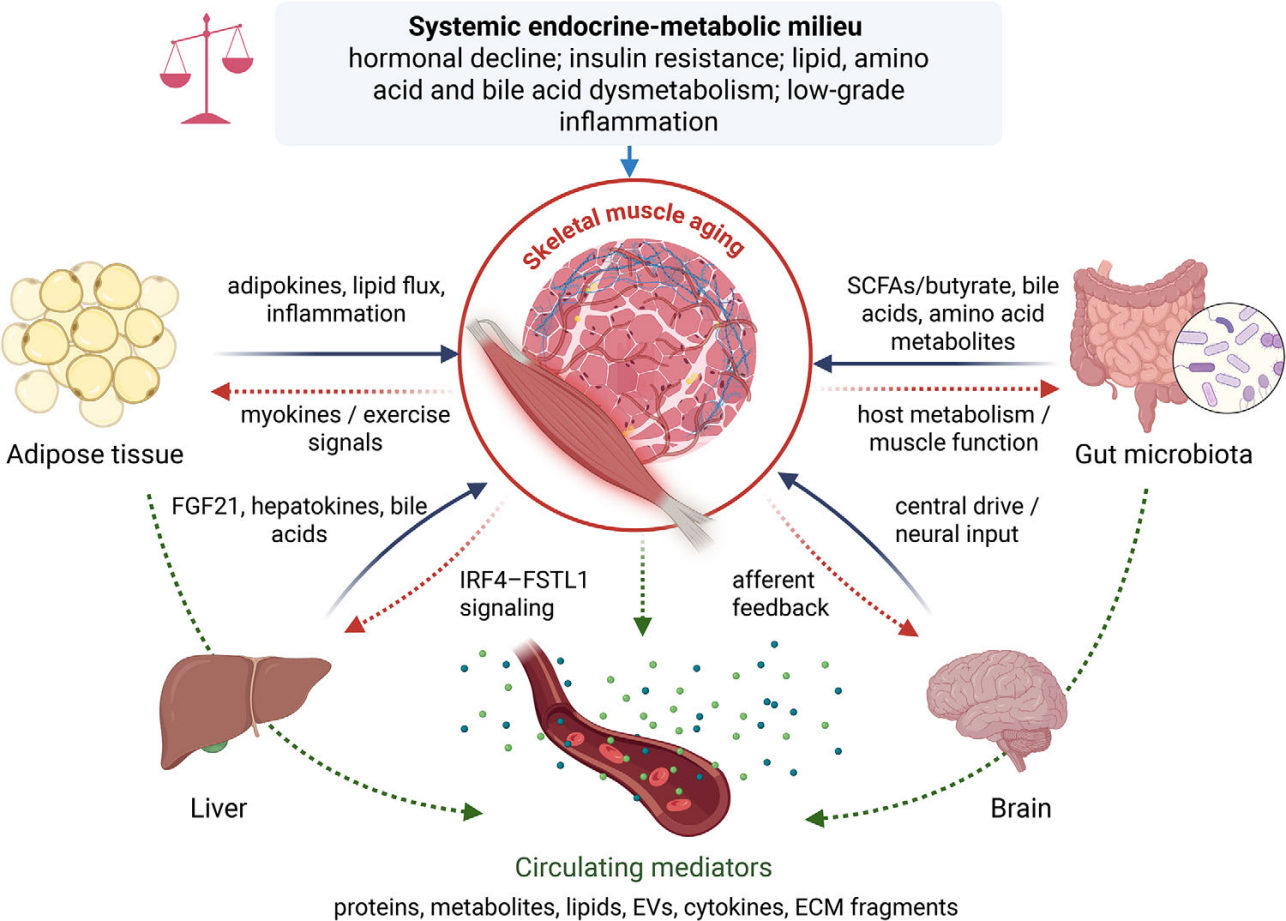
Systemic regulation and interorgan crosstalk in skeletal muscle aging. Skeletal muscle aging is shaped by systemic endocrine, metabolic, inflammatory, microbial, and neural signals. Hormonal decline, insulin resistance, lipid, amino acid, and bile acid dysmetabolism, and low‐grade inflammation create a systemic milieu that modifies muscle anabolic sensitivity, mitochondrial quality control, immune tone, regeneration, and fibrosis. Adipose tissue influences muscle through adipokines, lipid flux and inflammatory mediators, whereas muscle‐derived myokines and exercise‐related signals feedback to adipose metabolism. The liver‐muscle axis involves hepatokines, bile acids and defined pathways such as FGF21 and IRF4‐FSTL1 signaling. Gut microbiota‐derived metabolites, including SCFAs/butyrate, bile acids and amino acid metabolites, link gut ecology to muscle metabolism and function. Brain–muscle communication adds neural input, central drive and afferent feedback. Circulating proteins, metabolites, lipids, extracellular vesicles, cytokines and ECM fragments provide accessible biomarkers and potential therapeutic clues, but require interpretation within integrated clinical and functional contexts. Figure created with BioRender.com.

Overall, systemic regulation reframes skeletal muscle aging as an interorgan process mediated by identifiable signals. Exercise‐induced myokines and metabolites link muscle contraction to whole‐body adaptation; adipokines and lipid flux connect adipose dysfunction to muscle quality; FGF21 illustrates a receptor‐defined liver‐muscle endocrine pathway through FGFR1c/β‐Klotho and downstream ERK1/2/AMPK signaling; butyrate links gut ecology to muscle oxidative metabolism through HDAC inhibition and PGC‐1α‐centered epigenetic regulation; and neural‐behavioral factors connect muscle function with brain aging (Figure 3). This molecularly grounded view provides a stronger basis for biomarker‐guided stratification and mechanism‐matched interventions.

## 5 Therapeutic Interventions: From Foundational Strategies to Emerging Targeted Approaches

The therapeutic landscape of skeletal muscle aging is shifting from a simple mass‐building paradigm toward a broader effort to restore muscle quality, functional reserve, regenerative capacity, metabolic resilience, and neuromuscular control [207, 208, 209]. This shift is driven by two observations from recent translational research. First, regulatory and trial‐design discussions increasingly emphasize that sarcopenia trials require clinically meaningful endpoints rather than isolated changes in lean mass [207]. Second, recent pharmacotherapy reviews show that many candidate drugs produce pathway‐specific or body‐composition effects, but their functional benefit depends on patient selection, background care, and endpoint choice [208, 209]. Exercise and nutrition remain the foundation of management because they engage several aging mechanisms simultaneously, whereas targeted pharmacological and regenerative strategies are most informative when they are matched to the dominant biological defect. Thus, the key therapeutic question is no longer whether an intervention can increase muscle size or modify a molecular pathway. It is whether it improves strength, mobility, fatigue resistance, recovery from illness or disuse, and patient‐centered function in biologically heterogeneous older adults [207, 208, 209].

### 5.1 Exercise and Nutritional Interventions as the Foundation of Management

Exercise remains the most consistently supported intervention for age‐related muscle decline. A recent network meta‐analysis showed that exercise improves sarcopenia‐related outcomes in older people, supporting exercise as the most evidence‐based first‐line strategy rather than merely a general lifestyle recommendation [210]. Resistance training stimulates muscle protein synthesis, improves neuromuscular recruitment, increases strength, and can partially overcome anabolic resistance [210, 211]. The practical prescription perspective is also important: resistance exercise must be delivered with sufficient intensity, progression, supervision or adherence support, and adaptation to frailty or comorbidity; otherwise, its mechanistic potential may not translate into functional benefit [209].

Recent clinical trials further show that multicomponent exercise is often more relevant for older adults than isolated hypertrophy‐oriented training. In community‐dwelling older adults with sarcopenia, graded progressive home‐based resistance exercise combined with aerobic exercise improved strength, balance, flexibility, and cardiorespiratory fitness, indicating that feasible home‐based programs can target both muscle and mobility domains [212]. In older adults with impaired physical function but preserved muscle mass, combined exercise and nutrition improved gait speed, grip strength, and quality of life, highlighting that “functional sarcopenia” can respond even when low muscle mass is not the dominant entry criterion [213]. These studies support a shift from muscle quantity alone toward muscle health, where strength, mobility, balance, and function are treated as primary outcomes.

Nutrition is most effective when it complements exercise [213, 214]. Adequate total protein intake, leucine‐enriched protein, essential amino acids, vitamin D correction when deficient, and possibly creatine or β‐hydroxy‐β‐methylbutyrate may support anabolic responses and physical performance [211, 215, 216, 217]. The PROVIDE trial showed that a vitamin D and leucine‐enriched whey protein supplement improved measures of sarcopenia in older adults, supporting the concept that anabolic substrate and vitamin D status can modify response in selected populations [215]. Creatine supplementation combined with resistance training increased lean mass in older adults, but its value is best interpreted as an exercise adjunct rather than a stand‐alone antisarcopenia therapy [216]. Similarly, HMB supplementation improved strength, performance, or muscle quality in randomized studies of older adults with sarcopenia or postacute care needs, suggesting a role in catabolic or recovery‐prone contexts [211, 217]. The conclusion from these studies is not that nutrition alone reverses muscle aging. Rather, nutritional interventions appear most useful when they improve the anabolic environment in which exercise, recovery, and rehabilitation occur. For future trials of targeted drugs, exercise level, protein intake, vitamin D status, weight change, and rehabilitation exposure should be measured or standardized. Otherwise, it becomes difficult to determine whether a pharmacological agent failed biologically or whether the background environment prevented response.

### 5.2 Pharmacological Strategies Targeting Muscle Aging Mechanisms

Pharmacological interventions aim to target biological defects that contribute to muscle aging, including mitochondrial dysfunction, impaired anabolic signaling, senescence, inflammation, and defective repair [218, 219]. Their value lies in mechanistic specificity, but recent reviews emphasize a recurring limitation: skeletal muscle aging rarely results from a single dominant pathway [208, 219]. A drug that improves one mechanism may fail clinically if the patient's functional limitation is instead driven by denervation, fibrosis, inactivity, insufficient nutrition, systemic disease, or poor motor control. Therefore, the strongest pharmacological studies are those that link a defined mechanism to an appropriate population and endpoint.

#### 5.2.1 Mitochondria‐Targeted Interventions

Mitochondria‐targeted interventions are designed to improve energy metabolism, redox balance, mitochondrial turnover, and stress resilience [220, 221]. Recent studies have made this field more concrete by moving beyond general antioxidant concepts toward defined mitochondrial quality‐control pathways. Trigonelline was identified as a nicotinamide adenine dinucleotide (NAD^+^) precursor that is reduced in human sarcopenia and improves muscle function during aging, linking a circulating dietary/metabolic molecule to NAD^+^ availability, mitochondrial function, and muscle performance [221]. This study connects human sarcopenia‐associated metabolic deficiency with mechanistic validation in aging models, rather than treating NAD^+^ biology as a purely theoretical target. Urolithin A provides another example of translational progression. Initial preclinical work showed that Urolithin A induces mitophagy, extends lifespan in *C. elegans*, and increases muscle function in rodents [222]. Mechanistically, Urolithin A promotes mitochondrial turnover by activating the autophagy initiation machinery, including upregulation of the ULK1 complex and enhanced LC3 lipidation, thereby increasing autophagosome formation around damaged mitochondria [222]. A subsequent randomized clinical trial in older adults reported benefits on muscle endurance and mitochondrial health biomarkers, supporting the idea that mitophagy‐targeted interventions may improve endurance‐related outcomes even without large effects on muscle mass [223]. More recent work in trained male distance runners extends the question to performance and recovery contexts, indicating that mitochondrial‐targeted nutraceuticals may have endpoint‐specific effects depending on baseline fitness and physiological demand [224]. Elamipretide/SS‐31 illustrates a related but distinct strategy. In aged skeletal muscle mitochondria, elamipretide improved adenosine diphosphate (ADP) sensitivity through effects on adenine nucleotide translocator (ANT)‐dependent ADP uptake [220]. More broadly, SS‐31/elamipretide has been characterized as a cardiolipin‐targeting mitochondrial peptide that supports inner‐membrane bioenergetics [225]. A recent study further reported improved cardiac and skeletal muscle function during aging without detectable reversal of tissue epigenetic or transcriptomic age [226]. This distinction is important: mitochondrial therapeutics may improve organelle efficiency and function without globally reversing molecular aging signatures.

The translational implication is that mitochondrial interventions should not be judged primarily by appendicular lean mass. More appropriate endpoints include fatigue resistance, walking endurance, recovery after disuse or illness, mitochondrial biomarkers, metabolic flexibility, and muscle quality. They may be most effective in individuals whose phenotype is characterized by mitochondrial limitation, fatigue, poor recovery, or impaired exercise tolerance rather than by isolated low muscle mass [220, 221, 222, 223, 224, 226].

#### 5.2.2 Myostatin/Activin and Anabolic Approaches

The myostatin/activin pathway remains one of the most powerful biological targets for increasing muscle mass [227, 228, 229, 230, 231]. However, this area also provides the clearest evidence that increasing lean mass does not automatically produce proportional functional improvement. In community‐dwelling older adults with sarcopenia, bimagrumab, an activin type II receptor blocker, increased lean mass but did not provide a clearly proportional functional advantage over optimized standard care [228]. At the pharmacodynamic level, activin type II receptor blockade prevents ligands such as myostatin/GDF8 and activin A from engaging ActRIIA/B receptor complexes, thereby reducing Smad2/3‐dependent catabolic transcription and relieving suppression of Akt/mTOR‐related anabolic signaling [232]. This trial is highly informative for the sarcopenia field because it shows that anabolic gain must be converted into strength, mobility, and motor performance to become clinically meaningful.

Newer studies suggest that the most promising use of activin/myostatin blockade may be context‐specific. In a preclinical GLP‐1 receptor agonist context, antibody blockade of activin type II receptors preserved skeletal muscle mass and enhanced fat loss during weight‐loss therapy [227]. More recently, bimagrumab with semaglutide has been tested in obesity as a body‐composition strategy, reframing activin blockade as a potential approach to preserve or increase lean mass during pharmacological weight reduction rather than simply as a primary sarcopenia drug [230]. This is an important new perspective because lean‐mass preservation during GLP‐1RA‐induced weight loss is a different therapeutic goal from reversing primary age‐related sarcopenia. Apitegromab provides another boundary case. Apitegromab targets latent myostatin activation and has shown motor‐function benefit in later‐onset spinal muscular atrophy in the TOPAZ phase II study and SAPPHIRE phase III trial [229, 230, 231]. These results support the therapeutic value of myostatin‐pathway modulation in neuromuscular disease, but they should not be directly extrapolated to general muscle aging, where denervation, fibrosis, inactivity, inflammation, and metabolic disease may dominate the phenotype.

#### 5.2.3 Senescence‐ and Inflammation‐Targeted Strategies

Senescence‐ and inflammation‐targeted strategies are attractive because aged muscle contains senescent niche cells, altered macrophage states, and chronic immune‐stromal signaling [233, 234, 235, 236]. Recent work has shifted this field from broad anti‐inflammatory suppression toward targeting specific senescent‐cell states and pathological immune‐stromal circuits. This distinction matters because inflammation is not uniformly harmful: early inflammatory responses are required for debris clearance and regeneration, and transient senescence‐like programs may participate in tissue remodeling [234, 235]. Several preclinical studies now provide more specific evidence. Intermittent fisetin supplementation improved physical function and reduced cellular senescence in aged skeletal muscle, with comparisons to genetic senescent‐cell clearance and synthetic senolytic approaches [233]. Deletion of senescence‐associated β‐galactosidase (SA‐β‐Gal^+^) cells using senolytics improved muscle regeneration in old mice, directly supporting the idea that senescent cells can impair regenerative capacity [234]. Inhibition of p53‐MDM2 binding with BI01 reduced senescent cell abundance and improved adaptive responses of aged skeletal muscle, suggesting that senescence modulation can influence hypertrophic or regenerative adaptation rather than only baseline aging markers [235]. The field is also moving toward cell‐state and circuit‐level mechanisms. A 2025 study reported that senescent macrophages can induce ferroptosis in skeletal muscle and accelerate osteoarthritis‐related muscle atrophy, identifying a macrophage‐myofiber paracrine injury axis rather than a nonspecific inflammatory background [236]. In vitro work showing that fisetin alleviates d‐galactose‐induced senescence in C2C12 myoblasts further links senomorphic effects to metabolic and gene‐regulatory changes in myogenic cells [237]. Together with single‐nucleus senescence profiling that nominated Maraviroc as a candidate intervention [13], these studies suggest that senescence‐targeted therapy should be designed around defined senescent populations, secretory programs, and downstream tissue effects. The evidence boundary is important. Most senescence‐targeted data in skeletal muscle aging remain preclinical, cellular, or target‐discovery based. These interventions should therefore not be presented as established sarcopenia therapies. Their translational value will depend on biomarkers of senescence burden, identification of the relevant cell types, dosing schedules that avoid impairing normal repair, and functional endpoints that distinguish true tissue restoration from nonspecific anti‐inflammatory effects.

### 5.3 Regenerative and Advanced Therapeutic Approaches

**FIGURE 4:**
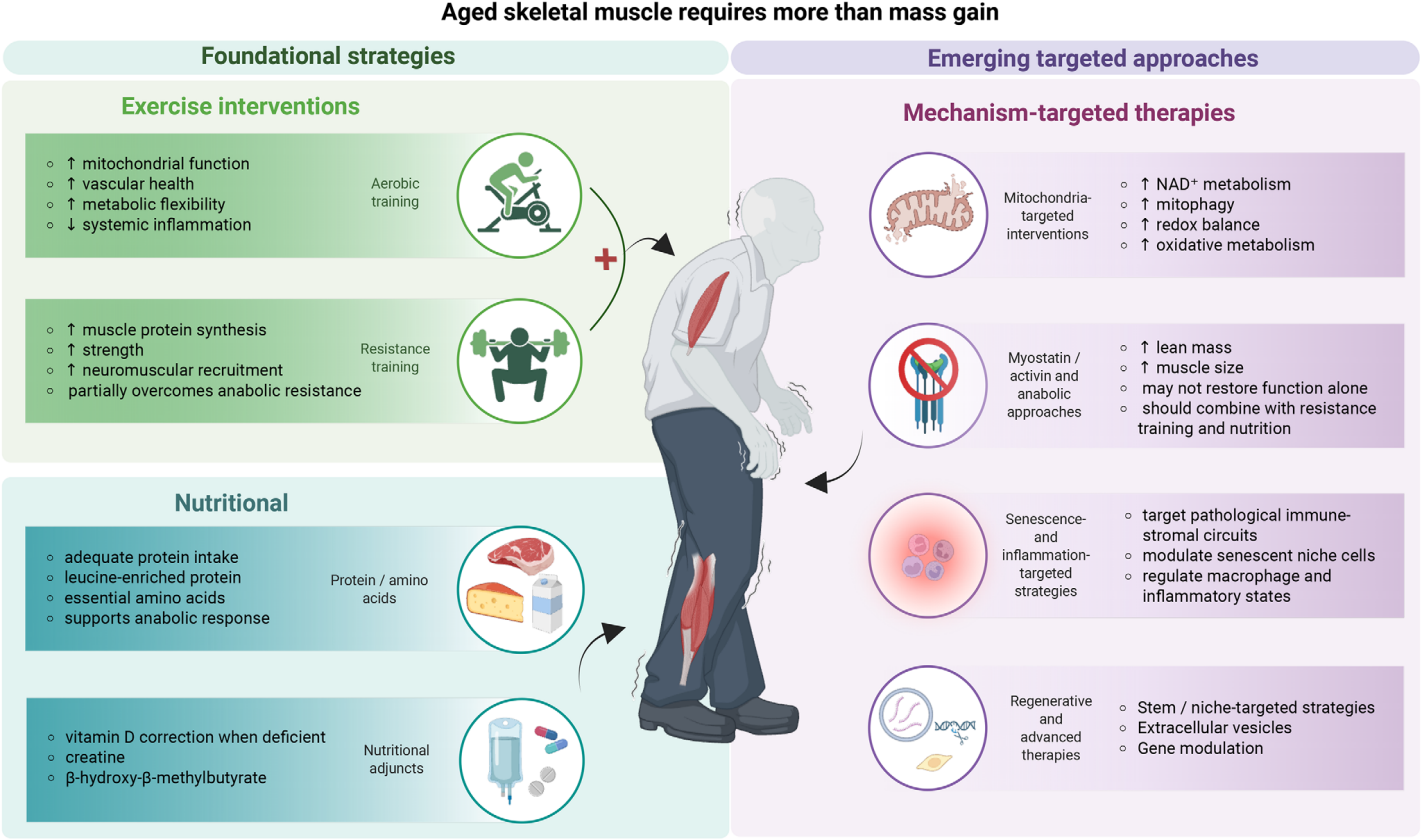
Therapeutic interventions for skeletal muscle aging. Aged skeletal muscle requires therapeutic approaches that restore muscle quality, functional reserve, regenerative capacity and neuromuscular control, rather than strategies focused only on mass gain. Foundational interventions remain central: aerobic exercise improves mitochondrial function, vascular health, metabolic flexibility and systemic inflammation, whereas resistance training stimulates muscle protein synthesis, strength and neuromuscular recruitment. Nutritional support, including adequate protein or amino acid intake, leucine‐enriched protein, vitamin D correction, creatine and β‐hydroxy‐β‐methylbutyrate, may improve the anabolic environment for exercise, recovery and rehabilitation. Emerging targeted approaches include mitochondria‐directed therapies, myostatin/activin and anabolic strategies, senescence‐ and inflammation‐targeted interventions, and regenerative or niche‐modifying therapies using stem/niche‐targeted strategies, extracellular vesicles or gene modulation. Together, these interventions support a shift toward mechanism‐matched, function‐centered strategies for preserving muscle health during aging. Figure created with BioRender.com.

Regenerative strategies aim to restore repair capacity rather than simply slow degeneration. Recent work has moved this field away from the idea of simply adding stem cells and toward engineering or rejuvenating the aged niche. This shift is necessary because aged muscle fails to regenerate not only due to satellite‐cell dysfunction, but also because the surrounding microenvironment is inflammatory, fibrotic, metabolically stressed, mechanically altered, poorly vascularized, or denervated [238, 239, 240]. Biomaterial studies illustrate how the niche can control regenerative competence. On‐demand stiffening and softening hydrogels revealed that skeletal muscle stem cells retain a mechanical memory, showing that prior exposure to matrix stiffness can shape later stem‐cell behavior [238]. Bioactive nanofiber‐hydrogel composites have also been designed to regulate the regenerative microenvironment after volumetric muscle loss, emphasizing that successful repair requires coordinated control of structural support, inflammation, vascularization, and myogenesis [239]. These studies support the idea that mechanical and biomaterial cues are not passive scaffolds but active regulators of regenerative outcome. Extracellular vesicle‐based approaches provide a second advanced strategy. Small extracellular vesicles from young plasma reversed age‐related functional declines in aged models by improving mitochondrial energy metabolism through PGC‐1α‐related mechanisms [241]. Myogenic exosome miR‐140‐5p was shown to modulate satellite‐cell activation and skeletal muscle injury repair, indicating that engineered or myogenic extracellular vesicle (EV) cargoes can directly influence regenerative cell behavior [242]. These findings suggest that EVs may serve as cell‐free modulators of aged muscle, but they also raise practical questions about source, cargo definition, dosing, biodistribution, durability, and manufacturing reproducibility. Other niche‐targeted approaches further broaden the regenerative framework. Skeletal muscle stem cells can regulate dystrophic niche function through a YY1–CCL5 axis, highlighting that MuSCs are not only myogenic precursors but also organizers of immune‐stromal communication [101]. Muscle‐derived extracellular matrix hydrogel promoted angiogenesis and neurogenesis in volumetric muscle loss, supporting the concept that functional regeneration requires vascular and neural reconstruction in addition to myofiber repair [240]. These studies are particularly relevant to aging because aged muscle often fails through combined deficits in stem cells, ECM, vascular support, and neuromuscular input (Table 2; Figure 4).

**TABLE 2:** Representative preclinical animal studies and clinical trials targeting skeletal muscle aging, organized by pathway and mechanism of action.

Targeted pathway	Intervention	Category	Evidence/trial stage	Context	Design	Objectives	Main response or preliminary findings	Trial registration	Status	Ref.
Mechanical loading, neuromuscular recruitment and anabolic adaptation	Resistance exercise	Exercise	Systematic review	Older adults with sarcopenia	Varied exercise modalities and durations	To compare exercise strategies for sarcopenia in older adults	Improved strength and physical function	N/A	N/A	[210]
Multicomponent exercise adaptation involving strength, balance and cardiorespiratory fitness	Resistance plus aerobic exercise	Exercise	Human RCT	Older adults	12‐week graded home‐based program	To evaluate the effects of individualized home‐based aerobic plus resistance exercise on physical fitness and cardiovascular function in older adults with sarcopenia	Improved strength, balance, flexibility, and fitness	ChiCTR1900027960	Completed	[212]
Exercise‐nutrition synergy for functional sarcopenia	Exercise plus nutritional intervention	Combined lifestyle intervention	Human RCT	Older adults with impaired function and preserved muscle mass	12‐week intervention	Evaluate whether a 12‐week exercise plus nutritional support program improves gait speed, grip strength, physical performance, and quality of life in older adults with functional sarcopenia	Improved gait speed, grip strength, and quality of life	KCT0008953	Completed	[213]
Exercise‐nutrition synergy affecting muscle quality and performance	Combined exercise and nutritional intervention	Combined lifestyle intervention	Systematic review	Frail and healthy older adults	Varied protocols	To evaluate whether combined exercise and nutrition interventions prevent or improve sarcopenia	Improved muscle quality, strength, and performance	N/A	N/A	[214]
Leucine‐sensitive protein synthesis and vitamin D‐related muscle function	Leucine‐enriched whey protein plus vitamin D	Nutrition	Human RCT	Older adults with sarcopenia	13‐week oral supplementation	Assess whether a whey protein‐, leucine‐, and vitamin D‐enriched supplement improves muscle strength and physical function in older adults with mobility limitations and low muscle mass	Improved muscle mass and lower‐extremity function	NTR2329	Completed	[215]
Phosphocreatine energy‐buffering system supporting training adaptation	Creatine plus resistance training	Nutrition plus exercise adjunct	Human RCT	Elderly adults	Low‐dose creatine with resistance training; 12 weeks	To assess whether creatine supplementation combined with resistance training improves lean mass and strength in older adults	Increased lean mass with resistance training	RBR‐7r7t52	Completed	[216]
HMB‐supported anabolic and anticatabolic response during resistance exercise	HMB plus resistance exercise	Nutrition plus exercise adjunct	Human RCT	Older adults with sarcopenia	HMB during resistance exercise; 12 weeks	To evaluate the effect of intervention on senile sarcopenia by HMB nutritional supplement intervention in senile sarcopenia patients	Improved strength, performance, and muscle quality	ChiCTR2000028778	Completed	[211]
HMB‐supported recovery of strength and physical performance	Calcium HMB plus resistance exercise	Nutrition plus exercise adjunct	Human RCT	Older/postacute sarcopenia context	3g/day Ca‐HMB with resistance exercise	Assess the effects of β‐HMB plus resistance training on muscle mass, strength, and physical performance in older postacute patients with sarcopenia	Improved strength and physical performance	NCT02679742	Completed	[217]
NAD^+^ metabolism and mitochondrial bioenergetics	Trigonelline	Mitochondria‐targeted metabolic intervention	Preclinical study	Human sarcopenia and aging models	Human metabolomics plus supplementation in aging models	Evaluate trigonelline as an NAD+‐boosting strategy to improve mitochondrial function and age‐related muscle decline	Improved mitochondrial function and muscle performance in aging models	N/A	N/A	[221]
Mitophagy and mitochondrial quality control	Urolithin A	Mitophagy activator/microbiota‐derived metabolite	Preclinical study	*C. elegans* and rodents; aging models	Urolithin A administration	Evaluate urolithin A as a mitophagy‐inducing strategy to improve mitochondrial and muscle function during ageing	Induced mitophagy and improved muscle function in aging models	N/A	N/A	[222]
Mitophagy‐linked mitochondrial health and muscle endurance	Urolithin A	Nutraceutical/mitophagy‐targeted intervention	Human RCT	Older adults aged 65–90 years	1000mg/day for 4 months	Assess whether daily urolithin A supplementation improves muscle bioenergetics, mitochondrial function, and physical performance in older adults	Improved muscle endurance and mitochondrial biomarkers	NCT03283462	Completed	[223]
ANT‐dependent mitochondrial ADP sensitivity	Elamipretide/SS‐31	Mitochondria‐targeted peptide	Preclinical study	Aged skeletal muscle	Elamipretide treatment in aged animals	Assess whether elamipretide improves mitochondrial ADP sensitivity and restores muscle and cardiac function during ageing	Improved mitochondrial ADP sensitivity and muscle force	N/A	N/A	[220]
Mitochondria‐targeted peptide therapy for age‐related muscle dysfunction	Elamipretide/SS‐31	Mitochondria‐targeted peptide	Preclinical study	Aged mice	Elamipretide treatment with functional and molecular aging readouts	Assess whether elamipretide treatment mitigates age‐related frailty, skeletal muscle dysfunction, and cardiac dysfunction in aged mice	Improved muscle function without molecular age reversal	N/A	N/A	[226]
Activin type II receptor signaling	Bimagrumab	Anabolic antibody/ActRII blockade	Human RCT; Phase 2	Older adults with sarcopenia and mobility limitation	Bimagrumab vs. placebo with nutrition and light exercise; 6 months	Assess whether bimagrumab improves physical function, skeletal muscle mass, and muscle strength in older adults with sarcopenia	Increased lean mass without clear functional benefit	NCT02333331	Completed	[228]
Activin type II receptor blockade combined with GLP‐1RA‐based obesity therapy	Bimagrumab with/without semaglutide	Anabolic antibody plus metabolic therapy	Human RCT; Phase 2	Adults with obesity	Bimagrumab, semaglutide or combination therapy; 72 weeks	Evaluate the efficacy and safety of bimagrumab alone or with semaglutide for weight loss while preserving lean/muscle mass in adults with overweight or obesity	Reduced fat mass with lean‐mass preservation	NCT05616013	Completed	[230]
Activin type II receptor blockade during GLP‐1RA‐driven weight loss	ActRII blockade plus GLP‐1RA context	Anabolic/body‐composition preserving strategy	Preclinical study	Mouse diet‐induced obesity model	ActRII antibody blockade alone or with GLP‐1RA	Assess whether ActRII blockade with bimagrumab preserves lean mass and enhances fat loss during GLP‐1 receptor agonist‐induced weight loss	Preserved muscle mass during GLP‐1RA‐induced weight loss	N/A	N/A	[227]
Latent myostatin activation blockade	Apitegromab	Myostatin‐targeted monoclonal antibody	Phase 2 clinical trial	Later‐onset SMA type2/3	TOPAZ phase II study	Assess whether SRK‐015/apitegromab improves motor function in patients with later‐onset spinal muscular atrophy type 2 or 3	Improved motor function in later‐onset SMA	NCT03921528	Completed	[229]
Latent myostatin activation blockade in SMA	Apitegromab	Myostatin‐targeted monoclonal antibody	Human RCT; Phase 3	Nonambulatory SMA type2/3	SAPPHIRE phase III trial; added to SMN‐targeted therapy	Assess whether apitegromab improves motor function and safety as adjunctive therapy in patients with later‐onset spinal muscular atrophy receiving nusinersen or risdiplam	Improved motor function in nonambulatory SMA	NCT05156320	Completed	[231]
Cellular senescence‐associated remodeling in aged skeletal muscle	Fisetin	Senolytic/senomorphic flavonoid	Preclinical study	Naturally aged mice	Intermittent fisetin supplementation	Assess whether intermittent fisetin supplementation reduces skeletal muscle senescence and improves frailty and grip strength during aging	Improved frailty and grip strength in aged mice			[233]
Senescent cell clearance/senolysis	Dasatinib plus quercetin	Senolytic combination	Preclinical study	Aged mice with impaired regeneration	Senolytic treatment around regenerative challenge	Assess whether dasatinib plus quercetin improves skeletal muscle regeneration and physical function in aged mice after injury	Improved regeneration and physical function in old mice	N/A	N/A	[234]
p53‐MDM2‐related senescence modulation and adaptive muscle remodeling	BI01	Senolytic/p53‐MDM2 inhibitor	Preclinical study	Old mice	BI01 treatment during regenerative or hypertrophic contexts	Assess whether BI01‐mediated p53 activation reduces senescent cell burden and enhances muscle regeneration and hypertrophy in aged mice	Reduced senescent cells and improved aged‐muscle adaptation	N/A	N/A	[235]
Cell‐type‐specific SASP and CCR5‐related senotherapeutic targeting	Maraviroc candidate identified through multiomics	Senomorphic/drug‐repurposing strategy	Preclinical study	Aging human skeletal muscle/sarcopenia‐related senescence	Senescence atlas and therapeutic target discovery	Map cellular senescence in ageing human skeletal muscle and evaluate Maraviroc as a senotherapeutic strategy for sarcopenia	Identified senescent‐cell states and Maraviroc as a candidate intervention			[13]
Senescent macrophage‐ferroptosis paracrine axis	Targeting senescent macrophage‐myofiber circuit	Immune‐stromal/antisenescent strategy	Preclinical study	Muscle atrophy associated with senescent immune remodeling	Analysis of senescent macrophage effects on muscle cells	Assess whether CoQ10 mitigates OA‐related muscle atrophy by suppressing ferroptosis	Revealed macrophage‐driven ferroptosis‐related muscle atrophy	N/A	N/A	[236]
Circulating sEV‐PGC‐1α‐mitochondrial energy metabolism axis	Young plasma small extracellular vesicles	Cell‐free regenerative/rejuvenation strategy	Preclinical study	Aged mice with aging‐related functional decline	Young plasma‐derived sEV administration	To test whether young plasma‐derived sEVs mediate systemic rejuvenation in aged mice	Improved age‐related functional decline via mitochondrial metabolism	N/A	N/A	[241]
Exosome‐mediated miRNA regulation of satellite‐cell activation and muscle repair	Engineered myogenic exosomes carrying miR‐140‐5p inhibitor	Engineered vesicle/RNA cargo therapy	Preclinical study	Mouse and myogenic cell injury/repair models	Engineered exosome delivery after muscle injury	To examine whether miR‐140‐5p‐inhibited myogenic exosomes promote skeletal muscle repair by activating satellite cells	Promoted satellite‐cell activation and injury repair	N/A	N/A	[242]
ECM stiffness, mechanotransduction and MuSC mechanical memory	On‐demand stiffening/softening hydrogel	Biomaterial/mechanical niche intervention	Preclinical study	Mouse and ex vivo MuSC systems	Tunable hydrogel exposure to dynamic mechanical environments	To determine whether microenvironmental stiffness induces a lasting mechanical memory that impairs MuSC regenerative function	Revealed stiffness‐dependent MuSC mechanical memory	N/A	N/A	[238]
ECM‐mediated angiogenic and neurogenic regenerative niche remodeling	Muscle‐derived ECM hydrogel	Biomaterial/ECM niche therapy	Preclinical study	Mouse volumetric muscle loss model	ECM hydrogel applied to severe muscle loss injury	To determine whether microenvironmental stiffness induces a lasting mechanical memory that impairs MuSC regenerative function	Enhanced angiogenesis, innervation, and tissue remodeling	N/A	N/A	[240]
MuSC‐derived YY1‐CCL5 signaling and immune‐stromal niche crosstalk	Modulation of MuSC‐derived niche signaling	Gene/niche‐targeted intervention	Preclinical study	Dystrophic muscle niche/chronic muscle pathology	Genetic and mechanistic analysis of MuSC‐derived signals	To determine how MuSC‐derived YY1‐CCL5 signaling regulates macrophage‐FAP niche interactions and dystrophic muscle pathology	Defined YY1‐CCL5 regulation of MuSC‐immune‐stromal niche crosstalk	N/A	N/A	[101]

### 5.4 Translational Barriers and Trial Design Priorities

The therapeutic pipeline reveals a persistent gap between preclinical efficacy and clinical benefit. Animal studies and early mechanistic work show promising effects of mitochondrial interventions, activin/myostatin blockade, senescence modulation, extracellular vesicles, biomaterials, and niche‐targeted approaches [101, 220, 221, 223, 228, 233, 235, 240, 241]. However, recent translational reviews emphasize that sarcopenia pharmacotherapy remains “lost in translation” when mechanisms, populations, and endpoints are not aligned [243, 244]. In humans, the most reproducible functional benefits still come from exercise, nutritional optimization, and multicomponent lifestyle‐based interventions [210, 211, 212, 213, 214, 217].

One barrier is population heterogeneity. Clinical trials may group together healthy older adults, frail individuals, patients with primary sarcopenia, people with obesity or diabetes, postacute care patients, weight‐loss therapy users, and patients with neuromuscular disease [207, 212, 213, 217, 229, 231]. These groups do not share the same biology. A therapy that preserves lean mass during GLP‐1RA‐associated weight loss may not reverse denervation‐driven weakness [227, 230]. A myostatin‐pathway drug that benefits SMA cannot be assumed to benefit primary age‐related sarcopenia [229, 231]. A mitochondrial intervention may improve endurance or biomarkers but have limited impact if the dominant limitation is fibrosis, neural control, or inactivity [220, 221, 222, 223, 226].

A second barrier is endpoint mismatch. The 2025 trial‐endpoint discussion and biomarker consensus both support a broader evaluation framework that includes functional, structural, and humoral dimensions rather than lean mass alone [207, 245]. Appendicular lean mass, grip strength, gait speed, chair‐stand performance, 6 min walk distance, falls, patient‐reported mobility, imaging‐based muscle quality, biopsy markers, and circulating biomarkers capture different dimensions of muscle health [245, 246]. A mitochondrial therapy should be evaluated with endurance, fatigue resistance, recovery, and mitochondrial biomarkers; an anabolic therapy should demonstrate conversion of mass into strength or mobility; an NMJ‐oriented approach should include neuromuscular measures; and an antifibrotic or niche therapy should include imaging or tissue markers of muscle quality.

The most recent consensus trends also support a shift from sarcopenia as a narrow diagnostic label toward muscle health and precision intervention. AWGS 2025 explicitly shifts attention from sarcopenia alone to muscle health, while precision‐health perspectives argue for personalized interventions based on biological and functional profiles [247, 248]. Prospective studies further show that physical performance and body composition have predictive value for metabolic outcomes, reinforcing the need to treat muscle function as a systemic health indicator rather than a purely musculoskeletal endpoint [249, 250].

Future trials should therefore combine mechanism‐matched enrollment, optimized background care, and function‐centered endpoints. Exercise exposure, protein intake, vitamin D status, weight change, comorbidities, medications, and rehabilitation context must be measured because they can determine treatment response [207, 211, 217]. Circulating biomarkers, imaging‐based muscle quality, functional testing, and omics‐derived signatures are beginning to define biologically distinct muscle‐aging trajectories, but they must be integrated into trial design rather than added only as exploratory endpoints [245, 247]. Therapeutic development should move from testing “anti‐sarcopenia drugs” in broad populations toward testing mechanism‐specific interventions in biologically defined subgroups, with outcomes that reflect real functional benefit.

## 6 Conclusions and Future Perspectives

Skeletal muscle aging is best understood as a decline in tissue resilience rather than as loss of muscle mass alone. This resilience depends on the coordinated maintenance of proteostasis, mitochondrial function, immune resolution, stem cell competence, ECM mechanics, neuromuscular control, and systemic metabolic support [243, 244, 246, 247]. The next challenge is to convert this multiscale understanding into clinically useful stratification and intervention strategies. At the same time, a model that includes many interacting mechanisms may be difficult to apply at the bedside. Future work should therefore distinguish broadly informative mechanisms from those that are most actionable, and identify a smaller number of biological bottlenecks that can be prioritized for initial intervention. Chronic low‐grade inflammation, for example, may act less as an isolated pathway than as an amplifier of mitochondrial stress, impaired regeneration, fibrosis, denervation, and systemic metabolic dysfunction.

### 6.1 Integrating Multiscale Mechanisms Into Precision Interventions

A precision framework for muscle aging should classify older adults according to dominant biological constraints rather than by muscle mass alone. Older adults with similar appendicular lean mass may differ markedly in their dominant biology: some may show anabolic resistance and inactivity‐related atrophy, whereas others may be driven by mitochondrial dysfunction, inflammatory niche remodeling, fibrosis, denervation, metabolic disease, or impaired regeneration. Treating these individuals as a single clinical group risks diluting therapeutic effects and obscuring mechanism‐specific benefit. A useful approach is mechanism‐based stratification. Mitochondria‐targeted strategies may be most appropriate for individuals with impaired metabolic flexibility, fatigue resistance, or mitochondrial quality control. Senescence‐ or inflammation‐targeted therapies may require evidence of pathological senescence‐associated secretory phenotype (SASP) activity or immune‐stromal activation. Antifibrotic or FAP‐directed approaches may be most relevant when muscle quality is limited by ECM remodeling or myosteatosis. NMJ‐focused interventions may be needed when weakness exceeds what would be expected from muscle quantity alone. Anabolic therapies, meanwhile, may require adequate nutrition, resistance exercise, and preserved neuromuscular recruitment to translate increased mass into functional improvement. In this setting, biomarkers should serve as tools for biological interpretation rather than simple diagnostic labels. Circulating proteins, metabolites, inflammatory mediators, extracellular matrix fragments, imaging‐based muscle quality measures, functional tests, and tissue‐level omics can help link patient phenotype to dominant mechanism. The goal is not to replace clinical assessment with molecular profiling, but to combine them so that interventions can be matched to the biological defects most likely to limit function. To make this concept more operational, future studies could combine functional phenotyping with a small mechanism‐oriented biomarker panel. Such a framework should be considered hypothesis‐generating rather than diagnostic: a biological subgroup should be assigned only when functional performance, imaging features, and circulating markers point toward the same dominant constraint (Table 3).

**TABLE 3:** A hypothesis‐generating workflow for biomarker–functional stratification in skeletal muscle aging.

Step	Key action	Proposed implementation and interpretation
1	Define the dominant functional limitation	Interpret appendicular lean mass, grip strength, gait speed, chair‐rise performance, 6 min walk distance, fatigue resistance, and imaging‐derived muscle quality collectively, rather than relying on muscle mass alone
2	Add a mechanism‐oriented circulating biomarker panel	Candidate domains include CAF for NMJ remodeling; carnitine‐ and acylcarnitine‐related metabolites for mitochondrial substrate handling; amino acid metabolites, insulin‐resistance markers, myostatin, and activin A for anabolic–catabolic balance; ECM‐ or collagen‐turnover fragments for stromal remodeling; and SASP‐ or immune–stromal‐associated markers, such as IL‐6, CCL5/CCR5‐related signals, and GPNMB‐positive macrophage programs
3	Assign provisional biological subgroups when functional and molecular evidence converge	NMJ‐instability‐dominant: elevated CAF with weakness or neuromuscular fatigue disproportionate to muscle mass. Mitochondrial‐limited: reduced fatigue resistance, recurrent falls, or low walking endurance accompanied by mitochondrial energetic or carnitine‐related abnormalities. Senescence‐inflammatory niche: poor recovery and inflammatory features accompanied by senescence‐ or immune‐stromal‐associated markers. Fibrotic/FAP‐dominant: low muscle attenuation, myosteatosis, stiffness‐related limitation, or ECM/FAP‐associated signatures. Anabolic‐resistance/metabolic: low muscle mass and maximal strength accompanied by insulin resistance, amino acid imbalance, or elevated myostatin/activin‐related signals
4	Use the subgroups as research stratifiers rather than standalone diagnoses	Apply these provisional subgroups to enrich clinical trials for participants most likely to respond to mechanism‐matched interventions and to generate testable hypotheses for prospective validation

### 6.2 Future Directions for Translational Research

Several priorities follow from this framework. First, longitudinal human studies are needed to distinguish early drivers from late consequences or compensatory responses. Cross‐sectional atlases and multi‐omics studies have defined important cell states and pathways, but they cannot fully establish when these changes emerge, whether they predict functional decline, or whether they are reversible. Longitudinal designs that integrate tissue profiling, circulating biomarkers, imaging, and repeated functional assessment will be essential [245]. Spatial transcriptomic and related spatial omics approaches should also be incorporated where possible, because they can localize senescent‐like stem or progenitor states, immune‐stromal interactions, and matrix‐remodeling programs within defined injury, fibrotic, inflammatory, or neuromuscular niches of aged muscle. Second, future studies should better align experimental models with human aging. Aged animal models remain indispensable for mechanistic testing, but human sarcopenia develops in the context of sex differences, inactivity, obesity, diabetes, chronic inflammation, multimorbidity, polypharmacy, nutritional variability, and behavioral constraints. Preclinical studies that incorporate these modifiers may improve translational relevance. Third, clinical trials should move beyond single‐dimensional endpoints. Lean mass, grip strength, gait speed, chair‐stand performance, endurance, imaging‐derived muscle quality, biopsy‐based molecular signatures, falls, and patient‐reported mobility capture different aspects of muscle health [248, 249, 251]. Mechanism‐targeted therapies should be evaluated with endpoints that reflect their expected biological action. For example, a mitochondrial intervention should not be judged only by appendicular lean mass, and an anabolic agent should not be considered successful unless mass gain translates into strength or mobility benefit [246, 250]. Finally, future interventions will likely need to be rational combinations. Exercise and nutrition should remain foundational because they coordinate multiple adaptive systems. Targeted pharmacological, senescence‐directed, mitochondrial, anabolic, regenerative, or neuromuscular approaches may be most effective when layered onto optimized background care and selected according to biological phenotype. In this sense, the future of skeletal muscle aging research lies not in finding a universal antisarcopenia therapy, but in building precision strategies that restore tissue resilience, functional reserve, and systemic coordination in biologically defined older adults.

## Author Contributions

Y.M.H. supervised and conceptualized the manuscript. T.L. drafted the main text and conducted the literature analysis. T.L. designed and illustrated all schematic figures. Y.M.H. provided critical feedback, revised the content for intellectual clarity, and contributed to the refinement of the manuscript. All authors reviewed and approved the final version of the manuscript.

## Funding

This work was supported by the National Scientific Foundation of China (No. 81870554; U22A20287).

## Ethics Statement and Consent to Participate

The authors have nothing to report.

## Conflicts of Interest

The authors declare no conflicts of interest.

## Data Availability

The authors have nothing to report.
